# Frequency‐adjusted borders ordinal forest: A novel tree ensemble method for ordinal prediction

**DOI:** 10.1111/bmsp.12375

**Published:** 2024-12-08

**Authors:** Philip Buczak

**Affiliations:** ^1^ Department of Statistics TU Dortmund University Dortmund Germany; ^2^ Research Center Trustworthy Data Science and Security UA Ruhr Dortmund Germany

**Keywords:** machine learning, ordinal forest, ordinal prediction, random forest

## Abstract

Ordinal responses commonly occur in psychology, e.g., through school grades or rating scales. Where traditionally parametric statistical models like the proportional odds model have been used, machine learning (ML) methods such as random forest (RF) are increasingly employed for ordinal prediction. With new developments in assessment and new data sources yielding increasing quantities of data in the psychological sciences, such ML approaches promise high predictive performance. As RF does not inherently account for ordinality, several extensions have been proposed. A promising approach lies in assigning optimized numeric scores to the ordinal response categories and using regression RF. However, these optimization procedures are computationally expensive and have been shown to yield only situational benefit. In this work, I propose Frequency‐Adjusted Borders Ordinal Forest (fabOF), a novel tree ensemble method for ordinal prediction forgoing extensive optimization while offering improved predictive performance in simulation and an illustrative example of student performance. To aid interpretation, I additionally introduce a permutation variable importance measure for fabOF tailored towards ordinal prediction. When applied to the illustrative example, an interest in higher education, mother's education, and study time are identified as important predictors of student performance. The presented methodology is made available through an accompanying R package.

## 1 INTRODUCTION

Ordinal data are an everyday occurrence in psychology. School grades are strictly speaking ordinal, rating scales used in questionnaires produce ordinal responses, and raters award ordinal scores to performance in tasks or assessments. In statistical analyses, ordinal responses have long been modelled using parametric models. A well‐known parametric model class (containing, e.g., the proportional odds model) are cumulative models, which assume that the ordinal response results from an underlying latent variable that is only observable through thresholds (McCullagh, 1980). For a more general overview of parametric models for ordinal response data, the reader is referred to Tutz (2022). Similarly, ordinal regression can be approached from a Bayesian perspective. To this end, Johnson and Albert (1999) and Agresti and Hitchcock (2005) provide an overview of Bayesian ordinal regression models. For a tutorial on fitting ordinal regression models in the Bayesian framework, I refer the reader to Bürkner and Vuorre (2019). In the more traditional statistical modelling literature for psychology, efforts have been made to popularize statistical models for ordinal data (e.g., Bürkner & Vuorre, 2019; Sönning et al., 2024). At the same time, machine learning (ML) methods have been on the rise in these fields (e.g., Fife & D'Onofrio, 2022; Hilbert et al., 2021; Ulitzsch et al., 2022). These prediction‐oriented methods can easily handle large quantities of data, as are increasingly becoming available in the psychological sciences (Hilbert et al., 2021; Ulitzsch et al., 2022). Prediction is often of crucial interest for these fields, e.g., in aptitude‐fit assessment for trying to predict which students might benefit from what support. Further, there is a growing interest in predicting ordinal data, such as school grades (e.g., Costa‐Mendes et al., 2020) or creativity ratings (e.g., Buczak et al., 2022). These studies often rely on ML approaches that do not provide inherent support for ordinal responses such as random forest (RF; Breiman, 2001). To work around this limitation, different strategies have been proposed in the statistical and ML literature that enable the use of RF while accounting for the ordinal nature of the response. A common approach is assigning numeric scores and category borders to the ordinal response categories and using the scores as the outcome variable for training a regression RF and the category borders for predicting new observations (Buczak et al., 2024; Hornung, 2019). While in principle one could assign default scores of 1,2,…,k to the k ordinal response categories, it is unclear if such a choice is optimal and represents the assumed underlying latent variable well. Thus, ordinal forest (OF; Hornung, 2019) and the ordinal score optimization algorithm (OSOA; Buczak et al., 2024) both first aim to optimize the scores that are assigned to the ordinal categories. Split‐based ordinal forest (RFSp) proposed by Tutz (2021), on the other hand, does not rely on using numeric scores for the ordinal response categories and instead transforms the ordinal prediction task into a series of binary prediction tasks for which regular binary classification RFs are trained respectively. Using the individual RF models, cumulative probabilities for the ordinal response categories can be computed.

Apart from RF, other ML methods have been extended to ordinal prediction as well. Examples include boosting algorithms (Riccardi et al., 2014; Tutz & Hechenbichler, 2005), support vector machine (Chu & Keerthi, 2007; Herbrich, 1999), and neural networks (e.g., Cao et al., 2020; Cheng et al., 2008; Shi et al., 2023). While the ML literature generally offers a plethora of different methods, tree‐based methods have proven particularly effective for tabular data (Grinsztajn et al., 2022). As tabular data are common in psychology, and RF is currently among the most actively researched tree‐based methods for ordinal prediction (e.g., Buczak et al., 2024; Hornung, 2019; Janitza et al., 2016; Tutz, 2021), this work will focus mainly on the use of RF for ordinal prediction. A further practical benefit of RF is its relative robustness of hyperparameter choices, whereas other ML methods may require a computationally expensive hyperparameter tuning beforehand (see Probst et al., 2019).

In their comparison studies, Tutz (2021) and Buczak et al. (2024) only found slight differences regarding the predictive performance of the different RF‐based approaches. Further, Buczak et al. (2024) found the benefit of optimizing the scores assigned to the ordinal response categories as in OF and OSOA rather situational. The authors discuss possible reasons for the lack of consistent improvement such as the prediction procedure used in both methods. In this work, I introduce a novel prediction procedure for score‐based RFs as sketched in Buczak et al. (2024). Further, I point out that the direct link between scores and category borders (one is always determined by the other) in OF and OSOA can be another factor hindering improvement regarding predictive performance. To remedy this, I propose a novel score‐based RF method called *Frequency‐Adjusted Borders Ordinal Forest* (fabOF) which builds on the newly introduced prediction procedure and decouples the scores assigned to the ordinal response categories and the borders used for transforming the numeric predictions into ordinal categories. Instead of a computationally expensive optimization procedure as in OF and OSOA, fabOF chooses its borders using a heuristic based on the response category frequencies. Through simulation and an illustrative data example, I will demonstrate that fabOF improves upon the predictive performance of existing methods while requiring significantly less runtime than OF and OSOA, for example. Apart from predictive performance, explaining and interpreting results remains of crucial interest to psychological researchers, even in primarily predictive settings (Henninger et al., 2023). Particularly in the context of RF models, variable importance measures (VIMs) are a popular tool for quantifying the importance of individual covariates on model predictions (Molnar, 2022). While Janitza et al. (2016) proposed a VIM for ordinal prediction based on the ranked probability score (Epstein, 1969), their VIM is not applicable to the newly proposed fabOF, thereby necessitating an alternative solution. Therefore, I additionally introduce a custom VIM for fabOF that helps interpret the impact of individual covariates on fabOF model predictions.

The remainder of this paper is structured as follows. In the next section, I will present prior work on score‐based RF approaches to ordinal prediction in more detail. Subsequently, I will contribute to the literature by introducing the alternative prediction procedure as well as the newly proposed fabOF method and its custom VIM. Further, I will evaluate fabOF as well as its permutation VIM through simulation and showcase both using an illustrative data example. The work closes with a discussion and potential avenues for further research.

## 2 PRIOR WORK ON SCORE‐BASED ORDINAL PREDICTION WITH RANDOM FOREST

Assigning numeric scores to ordinal response categories is a common approach to extending existing ML algorithms to the ordinal case. Kramer et al. (2000) used numeric scores for predicting ordinal responses with regression trees. Piccarreta (2007), Archer (2010), and Galimberti et al. (2012) extended split criteria in classification trees based on numeric scores. The conditional inference tree framework by Hothorn et al. (2006) uses permutation tests to assess the association between the outcome variable and covariates. In their framework, which supports nominal, ordinal, and numeric responses, ordinal response categories are transformed into numeric scores that can be prespecified by the user. Janitza et al. (2016) have studied in detail the use of conditional inference forests for ordinal classification. In this work, a particular focus is placed on the score‐based RF methods OF (Hornung, 2019) and OSOA (Buczak et al., 2024).

Similar to the cumulative model, both methods assume that the ordinal response variable results from an underlying latent numeric variable. However, instead of the original numeric values, one can only observe a coarser version of the latent variable where individual observations take one of k ordinal categories. Both methods aim to approximate the latent variable by partitioning the [0,1] interval into k category‐specific subintervals characterized by the category borders $0\leq b_{1}<b_{2}<\text{⋯}<b_{k+1}\leq 1$. These category intervals are represented by a numeric score where for the *r*th category, OF and OSOA use the midpoint of the corresponding category interval $\left[b_{r},b_{r+1}\right)$ as its representative score $s_{r}$, i.e., $s_{r}=\frac{b_{r}+b_{r+1}}{2},r=1,\text{…},k$ (Buczak et al., 2024; Hornung, 2019). Finally, the numeric scores (and category borders) are transformed using the quantile function of the standard normal distribution (probit function) and used to fit a regression RF model. As a toy example, consider the following partition of the [0,1] interval into five category subintervals: [0,0.2),[0.2,0.4),[0.4,0.6),[0.6,0.8) and [0.8,1]. Using the midpoints of these intervals, the five categories would be represented by the numeric scores .1, .3, .5, .7, and .9 respectively. Transforming these scores (and category borders) using the probit function leads to numeric values in the interval (−∞,∞), which are in turn used to fit a regression RF that can output numeric predictions for new observations. Numeric predictions are translated back into ordinal categories using the category borders. Ignoring the probit transformation for the sake of the toy example, a numeric prediction of .15 for a given observation would fall into the first subinterval [0,0.2) and, thus, translate to the first ordinal category.

However, this toy example only showcases one possible partition of the [0,1] interval. In practice, it is not known which partition best represents the unknown underlying variable. Therefore, OF and OSOA employ an optimization procedure that aims to find the optimal partition regarding the predictive performance achieved with it. However, the two methods differ in their optimization strategy. OF starts by generating random sets of partitions. For each partition, a regression RF is trained using the numeric scores resulting from the respective partition as sketched in the toy example. The RF fits are evaluated using their out‐of‐bag (OOB) performance (i.e., for each observation only the trees which did not include the given observation in the training data are used for prediction and performance evaluation) as measured by Youden's index J (Youden, 1950). The partitions leading to the best performance are combined into a final partition that is used for fitting the final RF in a second step (Hornung, 2019). OSOA similarly evaluates partitions by their predictive performance using Youden's J. However, in contrast to OF, OSOA uses a non‐linear optimization algorithm that iteratively searches for an optimal partition. As such, the optimization procedure in OSOA can explore promising regions in the solution space instead of relying on a pregenerated set of possible partitions (Buczak et al., 2024).

For the non‐linear optimization, OSOA relies on the Sbplx optimizer from the NLopt library (Johnson, 2008), which is a variant of the Nelder–Mead method (Nelder & Mead, 1965). Relating back to the toy example, OSOA would select the partition [0,0.2),[0.2,0.4),[0.4,0.6),[0.6,0.8), and [0.8,1] as its starting point and would aim to iteratively pivot towards an optimal partition. Similar to OF, partitions are evaluated based on the predictive performance they achieve when training a regression RF and assessing its OOB performance. The internal optimizer traverses the space of possible partitions and aims to find the partition leading to the optimal value of Youden's index J. As such, OF and OSOA both aim to optimize the same performance measure, but with different means. Apart from their different optimization approaches, OF and OSOA are equivalent. In their comparison study, Buczak et al. (2024) found both methods to perform mostly similarly. Furthermore, the authors found the benefit of the score optimization to be situational as OF and OSOA could not consistently outperform a naive OF variant, which instead simply used the default scores 1,2,…,k and category borders 0.5,1.5,…,k+0.5.

Discussing potential improvements for OF and OSOA, Buczak et al. (2024) suggest using an alternative prediction procedure than the one currently employed. To predict new observations, both methods use the approach described in pseudocode in Algorithm 1. For all $n_{\text{pred}}$ observations to be predicted, each of the B trees is used to generate a numeric score prediction ${ŷ}_{ij}^{\text{num}}$, with $i=1,\text{…},n_{\text{pred}}$ and j=1,…,B. Using the category borders $b_{1},\text{…},b_{k+1}$ of the k response categories, the numeric score prediction ${ŷ}_{ij}^{\text{num}}$ can be transformed into a category label prediction ${ŷ}_{ij}^{\text{cat}}$. For each observation, the category label predictions from the B trees are aggregated via majority voting, resulting in the category label prediction for observation i, ${ŷ}_{i}^{\text{cat}}=\text{mode}({ŷ}_{i1}^{\text{cat}},\text{…},{ŷ}_{iB}^{\text{cat}})$. As this procedure first transforms the numeric score predictions into a category label and then aggregates all predicted category labels into a single category label prediction, it will be referred to as *transform‐first‐aggregate‐after* (TFAA) prediction in what follows.

Algorithm 1 **Transform‐first‐aggregate‐after (TFAA) prediction**

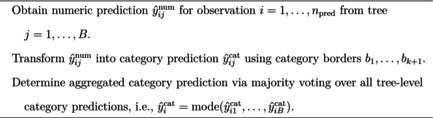

Buczak et al. (2024) argue that transforming numeric scores into category labels already at the tree level may limit the impact of the actual choices of the numeric scores. Instead, the authors suggest exploring an alternative prediction approach where the numeric predictions from the individual trees are first aggregated by averaging and then transforming the aggregated numeric prediction to a category label. I follow their suggestion and introduce *aggregate‐first‐transform‐after* (AFTA) prediction in the next section. Additionally, I address another limitation of OF and OSOA. For both methods, scores and category borders are directly linked since the scores are always determined as the midpoints of the category intervals. While always representing the categories by their midpoint may seem intuitive, it is not necessarily a decision based on predictive performance for the given data context. Therefore, separating category scores and borders from one another allows for additional flexibility. To this end, I propose fabOF, a novel score‐based RF method for ordinal prediction that builds on AFTA prediction and determines its category borders separately from its category scores in an adaptive way.

## 3 NEW CONTRIBUTIONS TO SCORE‐BASED ORDINAL PREDICTION WITH RANDOM FOREST

### 3.1 Aggregate‐first‐transform‐after prediction

AFTA prediction is described in pseudocode in Algorithm 2. In contrast to TFAA prediction, the tree‐level numeric score predictions ${ŷ}_{ij}^{\text{num}},i=1,\text{…},n_{\text{pred}},j=1,\text{…},B$ are first averaged to obtain an aggregated numeric score prediction, i.e.,

$${ŷ}_{i}^{\text{num}}=\frac{1}{B}\overset{B}{\underset{j=1}{\sum }}{ŷ}_{ij}^{\text{num}}.$$

By using the category borders, the aggregated score prediction can in turn be transformed into a category label prediction ${ŷ}_{i}^{\text{cat}}$. In this work, I investigate the use of AFTA prediction for existing methods such as OF and OSOA as well as for the newly proposed fabOF introduced in what follows.

Algorithm 2 **Aggregate‐first‐transform‐after (AFTA) prediction**

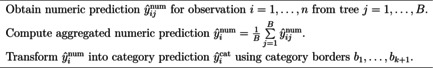

### 3.2 Frequency‐adjusted borders ordinal forest

The new fabOF method follows (naive) OF and OSOA in assigning scores to ordinal categories and using these to train a regression RF, which in turn outputs numeric score predictions for new observations that are transformed back into ordinal categories via category borders. However, in contrast to OF and OSOA, fabOF relies on AFTA prediction and separates the choice of scores and category borders. While in principle one could extend the optimization procedure of OF and OSOA to simultaneously optimize scores and category borders, such an approach would greatly increase the complexity of the optimization problem. Instead, fabOF employs a heuristic for deriving its category borders based on the distribution of the ordinal response categories. This design decision is motivated by the results obtained by Buczak et al. (2024) and Hornung (2019), both of whom indicate the response category distributions affect the predictive performance of ordinal prediction methods. I will demonstrate through simulation that combining AFTA prediction with the heuristic already leads to consistent improvements over existing methods in many cases while using the default scores 1,2,…,k. As such, fabOF does not make use of a computationally expensive optimization step.

Algorithm 3 describes fabOF in detail. After assigning the default scores 1,2,…,k to the ordinal response categories, a regression RF is trained using the respective covariates as predictors and the numeric score variable $Y^{\text{num}}$ as the target variable. From the RF model, numeric OOB predictions ${ŷ}_{i}^{\text{num}},i=1,\text{…},n$ for all observations are obtained. These numeric OOB predictions already constitute the aggregation step from the AFTA prediction approach since OOB predictions are generated at the tree level and then averaged into a combined OOB prediction for each observation. A key idea of fabOF is to employ a heuristic that determines the category borders in a way that matches the distribution of the predicted categories with the category distribution one would expect in the general population. To this end, fabOF computes the cumulative relative frequencies $\pi_{1},\pi_{2},\text{…},\pi_{k-1}$ of the ordinal response categories in the training data up to (but not including) category k. The inner set of category borders, i.e., $b_{2},\text{…},b_{k}$, are then determined by the quantiles $q_{\pi_{1}},q_{\pi_{2}},\text{…},q_{\pi_{k-1}}$ of the OOB predictions for probabilities $\pi_{1},\pi_{2},\text{…},\pi_{k-1}$. The bounding borders, i.e., $b_{1}$ and $b_{k+1}$, are set to 1 and k respectively, as they represent the minimum and maximum prediction values possible. By assigning the respective quantiles of the OOB predictions, the category borders are chosen such that the distribution of the predicted categories approximates the category distribution in the training data. This implicitly assumes that the training dataset is a suitable representation of the general population. The benefit of using the OOB predictions for determining the borders, instead of, for example, predictions obtained using the entire forest for each observation in the training set is that it reduces the risk of overfitting. As such, one can mimic the process of adjusting the borders to perform well on unseen data without having to set aside a separate set of data points solely to determine the appropriate borders. In the final step, fabOF returns the trained RF model as well as the category borders for predicting new observations. The proposed method is implemented in the R package fabOF, currently available via GitHub (https://github.com/phibuc/fabOF).

Algorithm 3 **Frequency‐Adjusted Borders Ordinal Forest (fabOF)**

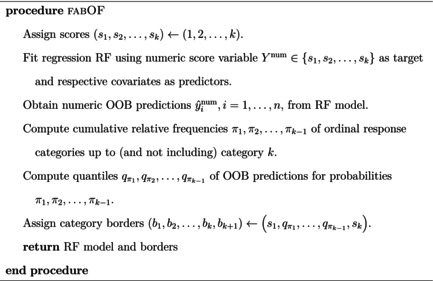

### Variable importance measure

While it can offer high predictive performance, a drawback of RF is its lack of interpretability (Henninger et al., 2023). However, when used, for example, in the context of predicting student attainment, it may not necessarily only be of interest to achieve high predictive model performance, but also to learn which factors, i.e., covariates, play a particularly influential role in the process. To aid interpretation of RF models in this regard, VIMs are often computed. VIMs aim to assess the importance of covariates by quantifying their impact on the predictive performance of a RF model (Molnar, 2022). A common class of VIMs are permutation VIMs (Breiman, 2001). To this end, the values of a given covariate are permuted and the resulting loss in predictive performance is measured. The logic behind permutation VIMs is that for important covariates, permuting the values leads to a larger loss in predictive performance as compared to less important covariates, because permutation voids the original information contained in the covariate (Molnar, 2022). As fabOF directly builds on RF, variable importance can be used to enhance the interpretability of fabOF as well. For ordinal prediction, Janitza et al. (2016) proposed a permutation VIM based on the ranked probability score (RPS; Epstein, 1969). Similarly, OF as implemented in the ordinalForest package (Hornung, 2022) offers the possibility of computing variable importance based on the RPS or on classification accuracy (i.e., ignoring ordinality). However, the RPS operates on the predicted ordinal response category probabilities, which are not available for fabOF. As such, a VIM relying on the RPS cannot be used for fabOF. Instead, I propose a custom permutation VIM for fabOF based on weighted Kappa as presented in pseudocode in Algorithm 4. It follows the classic flow of permutation VIMs as described in Fisher et al. (2019), where for each of the p covariates, the covariate values are permuted and the variable importance is computed as the difference in performance when using the original and the permuted data respectively. To account for the ordinality of the responses, weighted Kappa (Cohen, 1968) with linear weights (denoted by $\kappa^{\text{lin}}$) is used to measure predictive performance.

Algorithm 4 **Permutation VIM for fabOF**

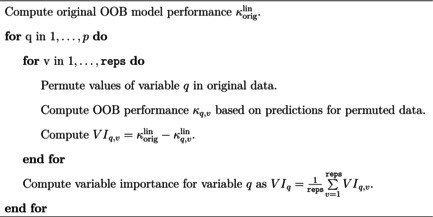

Typically, VIMs for RFs are computed at the tree level such that for each tree variable importance values for all covariates are obtained, which in turn are averaged, resulting in a single forest‐level importance value for each covariate. Since fabOF does not transform its numerical predictions into ordinal categories until the former are aggregated at the forest level, it is not feasible to compute variable importance based on an ordinal loss function at the tree level with fabOF. Computing variable importance at the forest level only, however, would mean that each covariate is permuted only once (as opposed to once per tree as in the typical approach), which could lead to unstable results (cf. Molnar, 2022). Therefore, fabOF's permutation VIM replicates the permutation process for each variable reps times where reps is a prespecified parameter modulating the trade‐off between stability of the VIM results and computation time.

## 4 SIMULATION STUDY 1: PREDICTIVE PERFORMANCE

### 4.1 Simulation setup

To evaluate fabOF, I performed a simulation study that compared fabOF with existing ordinal prediction methods such as (naive) OF, OSOA, RFSp, and a proportional odds model (referred to as CLM and specified with all linear main effects) as well as modified versions of (naive) OF and OSOA which employ AFTA prediction. For further computational details including software implementations and parameter choices, I refer the reader to Appendix A. The simulation setup was inspired by the simulation studies in Janitza et al. (2016) and Buczak et al. (2024). I simulated datasets with n∈{750,1500} observations and p=15 standard normally distributed and uncorrelated covariates. Six of the 15 covariates were influential, while the remaining were noise variables. To create diverse data scenarios, I created four different data‐generating processes (DGPs). To this end, I used two linear predictor functions $g_{1}(x)$ and $g_{2}(x)$ with 

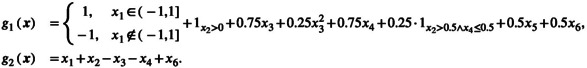

DGP 1 was simulated from a proportional odds model using $g_{1}(x)$ as the linear predictor. As such, the probability of the ordinal response Y taking at most category r=1,…,k was simulated as

$$P(Y\leq r|x)=\frac{\exp(\gamma_{r}+g_{1}(x))}{1+\exp(\gamma_{r}+g_{1}(x))},$$

where $\gamma_{r}$ is the respective threshold value with $-\infty <\gamma_{1}<\text{⋯}<\gamma_{k}=\infty$. For DGP 2, I added standard normal noise to $g_{1}(x)$, resulting in a linear regression model. The simulated numeric outcomes from the linear regression model were transformed into an ordinal response by binning. As binning is commonly used in practice, DGP 2 was designed to correspond to this frequent use‐case of ordinal prediction methods. DGP 3 was simulated from a proportional odds model where the linear predictor g(x) was obtained as a linear combination of $g_{1}(x)$ and $g_{2}(x)$, with

$$g(x)=0.6g_{1}(x)+0.4g_{2}(x).$$

For DGP 4, I replaced the normally distributed covariates $X_{5}$ and $X_{15}$ in DGP 1 by binary covariates. As DGPs 1–3 only included normally distributed covariates, the aim of DGP 4 was to check whether the presence of two binary covariates (one being influential, the other being noise) affects the performance of the prediction methods. Both binary covariates were simulated from a Bernoulli distribution with probability .5. To better reflect the original effect magnitude in DGP 1, I increased the effect of $X_{5}$ from .5 to 1 for DGP 4. The covariate $X_{15}$ remained a noise predictor for DGP 4. The effect sizes in $g_{1}(x)$ and $g_{2}(x)$ as well as the mixture component approach and mixture parameter choice were inspired by Janitza et al. (2016). Combining non‐linear and linear effects as in $g_{1}(x)$ was inspired by Buczak et al. (2024). I introduced further variety into the data generation using two different distribution patterns for the ordinal response categories, similar to Hornung (2019) and Buczak et al. (2024). By choosing specific threshold and binning values for the DGPs, I created a pattern of equally distributed categories, and a pattern with prominent middle categories (referred to as wide middle pattern) emerged. For more details on creating the patterns, see Appendix B.

The different prediction methods were evaluated using weighted Kappa with linear and quadratic weights (see Appendix C for more details) as well as Kendall's rank correlation (Kendall, 1945). Both have been used on ordinal prediction (e.g., Ben‐David, 2008; Buczak et al., 2024; Hornung, 2019). To this end, $\frac{2n}{3}$ observations were used to fit the prediction model while the remaining observations were used for evaluating the model on unseen data. Since fabOF determines its category borders based on the frequencies of the ordinal response categories in the training data, it works on the assumption that the training data are a suitable representation of the general population data. To study how fabOF reacts to a violation of this assumption, I considered two different sampling procedures in the simulation. After generating an initial sample of 10,000 observations (based on the DGP and response distribution pattern as specified by the respective simulation condition), I either drew a category‐stratified subsample or a random subsample without stratification of size $\frac{2n}{3}$ for the training set. The test set of $\frac{n}{3}$ observations was sampled with category stratification in both cases. This created two scenarios, where for the first scenario both training and test sets were a suitable representation of the general population, while for the second scenario the category distribution in the training set was (potentially) a misrepresentation of the category distribution in the population sample. All conditions were simulated with 1000 replications. As the development of the fabOF R package began at a later stage, a prototype implementation for fabOF was used in the simulation. This prototype implementation uses the exact same routine as the internal functions of the fabOF R package and differs only in its user interface and input handling. The prototype implementation is available from the corresponding OSF repository of this work at https://osf.io/fn8bg/.

### 4.2 Simulation results

In the following section, I will present the results from the simulation for each DGP. From the simulation parameters, the number of categories and the sampling procedure did not affect the results, while the impact of an increased number of observations was mostly limited to reducing the variability of the results as well as increasing the disparity between the CLM and the RF‐based methods. Therefore, I will only show results for simulation scenarios with n=750 observations, k=5 categories, and stratified sampling of the training sets in the following due to space reasons. For the remaining results, I refer to the Supporting Information.

#### Results for DGP 1

**FIGURE 1:**
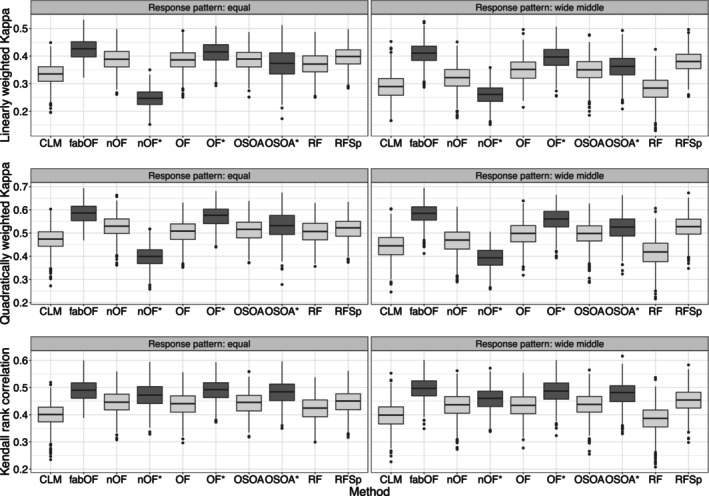
Predictive performance of all methods and modifications for data simulated from DGP 1 with n=750, k=5, and stratified sampling for both response category distribution patterns. Approaches using aggregate‐first‐transform‐after prediction indicated through dark grey coloured boxplots with additional asterisk indicating modification of existing method. CLM, cumulative link model (proportional odds); fabOF, Frequency‐Adjusted Borders Ordinal Forest; nOF, naive ordinal forest; OF, ordinal forest; OSOA, ordinal score optimization algorithm; RF, random forest; RFSp, split‐based ordinal forest.

Figure 1 shows the results for DGP 1 for the two response category distribution patterns. For weighted Kappa with linear and quadratic weights, fabOF generally reached the best performance alongside OF with AFTA prediction. Overall, the existing ordinal prediction methods performed similarly in the case of equally distributed response categories, while finer differences emerged for the wide middle pattern. For the latter case, all existing methods except RFSp suffered from the imbalanced data scenario. fabOF, on the other hand, held up quite well and gained more ground on most of the other methods. When Kendall's rank correlation was used as the performance measure, the differences between the individual methods (and modifications) were more subtle. For both response category distribution patterns, fabOF as well as AFTA‐modified OF and OSOA tended to achieve slightly higher correlation scores than the remaining methods. Overall, the effect of AFTA prediction was rather mixed. While it increased the performance of OF and tended to do so as well for OSOA, the effect direction for naive OF was dependent on the performance measure.

#### Results for DGP 2

**FIGURE 2:**
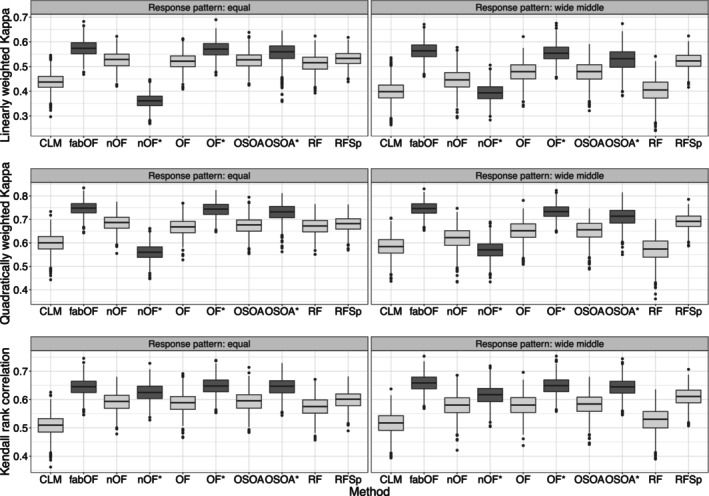
Predictive performance of all methods and modifications for data simulated from DGP 2 with n=750, k=5, and stratified sampling for both response category distribution patterns. Approaches using aggregate‐first‐transform‐after prediction indicated through dark grey coloured boxplots, with additional asterisk indicating modification of existing method. CLM, cumulative link model (proportional odds); fabOF, Frequency‐Adjusted Borders Ordinal Forest; nOF, naive ordinal forest; OF, ordinal forest; OSOA, ordinal score optimization algorithm; RF, random forest; RFSp, split‐based ordinal forest.

Figure 2 displays the results for DGP 2 for which, in contrast to DGP 1, the ordinal response was generated by binning numeric responses simulated from a linear regression model. Overall, these results mirror the results obtained for DGP 1. This is not surprising as the effect structure from DGP 1 carried over to DGP 2, and the only difference between the two DGPs was the model used for simulating the outcome. fabOF generally led to the best performance for weighted Kappa with linear and quadratic weights as well as Kendall's rank correlation. The differences were more apparent for weighted Kappa and the wide middle response category distribution pattern, while they were smaller for Kendall rank correlation scores. The AFTA prediction modified versions of OF and OSOA trailed only slightly behind fabOF and were also on par in some scenarios. While AFTA prediction benefited the predictive performance of OF and OSOA for all measures, it notably decreased the performance of naive OF for weighted Kappa and slightly increased it for Kendall's rank correlation.

#### Results for DGP 3

**FIGURE 3:**
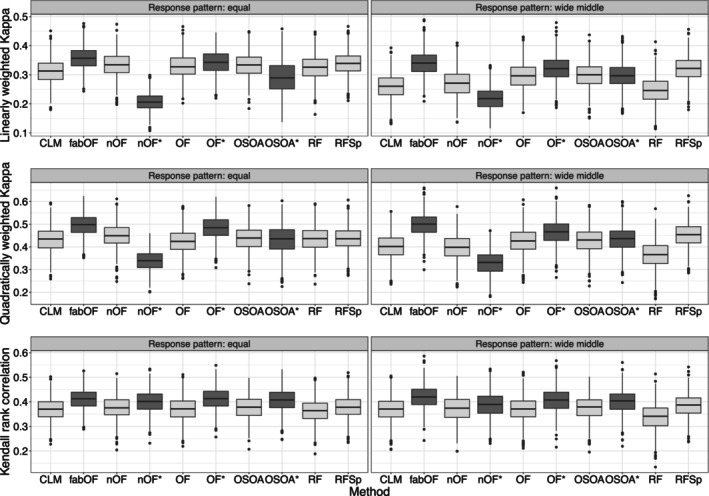
Predictive performance of all methods and modifications for data simulated from DGP 3 with n=750, k=5, and stratified sampling for both response category distribution patterns. Approaches using aggregate‐first‐transform‐after prediction indicated through dark grey coloured boxplots, with additional asterisk indicating modification of existing method. CLM, cumulative link model (proportional odds); fabOF, Frequency‐Adjusted Borders Ordinal Forest; nOF, naive ordinal forest; OF, ordinal forest; OSOA, ordinal score optimization algorithm; RF, random forest; RFSp, split‐based ordinal forest.

Figure 3 shows the results for DGP 3 in which the outcome was simulated through a mixture distribution. Compared to DGP 1 and DGP 2, the results were more homogeneous between methods while still adhering to the result patterns observed for DGPs 1 and 2. For weighted Kappa, the best performance was achieved by fabOF and AFTA‐modified OF. For Kendall's rank correlation, all methods were much closer in performance while still displaying a slight advantage for methods based on or modified with AFTA prediction. As before, only OF benefited consistently from the AFTA modification, while the effects were mixed and mostly subtle for OSOA. For naive OF, AFTA prediction greatly decreased predictive performance for weighted Kappa and slightly increased it for Kendall's rank correlation.

#### Results for DGP 4

**FIGURE 4:**
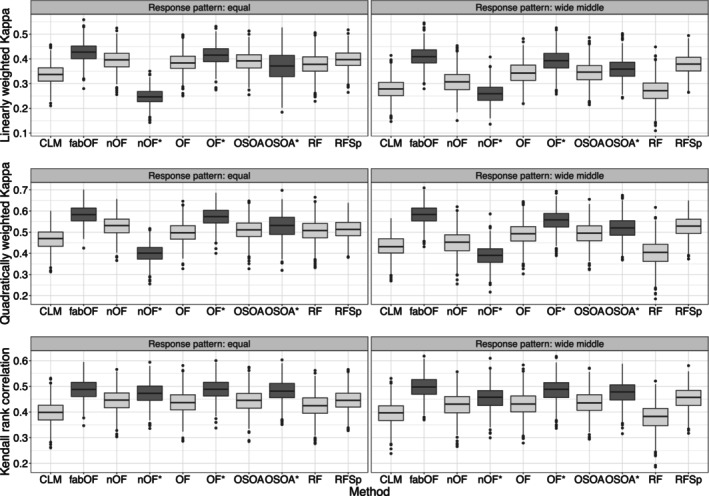
Predictive performance of all methods and modifications for data simulated from DGP 4 with n=750, k=5, and stratified sampling for both response category distribution patterns. Approaches using aggregate‐first‐transform‐after prediction indicated through dark grey coloured boxplots with additional asterisk indicating modification of existing method. CLM, cumulative link model (proportional odds); fabOF, Frequency‐Adjusted Borders Ordinal Forest; nOF, naive ordinal forest; OF, ordinal forest; OSOA, ordinal score optimization algorithm; RF, random forest; RFSp, split‐based ordinal forest.

Figure 4 shows the results for DGP 4 in which the effect structure of DGP 1 was modified to include two binary covariates, of which one was influential and one was noise. In comparison to the results for DGP 1, the inclusion of binary covariates did not affect the results to a notable degree. For weighted Kappa, fabOF and AFTA‐modified OF achieved the highest predictive performance, while for Kendall's rank correlation, AFTA‐modified OSOA was on par with the former two methods. For the wide middle response pattern, fabOF's heuristic led to slight performance advantages when compared to the pattern of equally distributed responses. Similar to the previous DGPs, AFTA prediction increased OF's predictive performance for all measures while the results were more mixed for OSOA and for naive OF.

### 4.3 Runtime comparison

Buczak et al. (2024) have shown that apart from the predictive performance, the runtime of ordinal prediction methods is important to consider as well, especially given the costly optimization procedures in OF and OSOA. To this end, I also compared the computation time required by the methods during the simulation in a similar fashion to that in Buczak et al. (2024). Note that since the computation of the simulation was carried out using a compute cluster, it cannot be guaranteed that the individual computations ran in perfectly comparable conditions (e.g., regarding the node selected by the cluster's workload manager or regarding the overall workload of the cluster at a given time). Further, all methods were restricted to only use one CPU for computation. This may have put methods relying on parallelization at a disadvantage. However, all RF‐based methods except for RFSp (which uses the randomForest package; Liaw & Wiener, 2002) used the RF implementation from the same package (i.e., ranger; Wright & Ziegler, 2017). In any case, the runtime results presented here should not be seen as an exact comparison, but rather as a ballpark estimate. As the choice of DGP, category distribution pattern or sampling strategy did not impact the runtime, I only show results for DGP 1 with equally distributed response categories and stratified sampling. Further, I limit the number of categories to five. While the other methods are not impacted by the choice of k, RFSp fits k‐1 RF models. As such, the number of categories is directly linked with the runtime of RFSp. For consistency with the presentation of the simulation results above, I selected k=5 for the runtime comparison here. For lower values of k, lesser runtimes are to be expected, and for higher values of k, longer runtimes of RFSp are to be expected.

**FIGURE 5:**
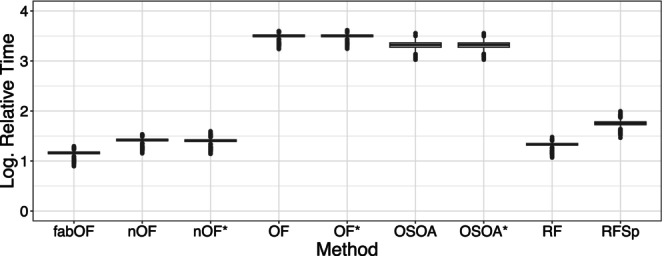
Logarithmized runtime (using base 10) relative to CLM for RF‐based methods. Modification of (naive) OF and OSOA with aggregate‐first‐transform‐after prediction indicated through asterisk. CLM, cumulative link model (proportional odds); fabOF, Frequency‐Adjusted Borders Ordinal Forest; nOF, naive ordinal forest; OF, ordinal forest; OSOA, ordinal score optimization algorithm; RF, random forest; RFSp, split‐based ordinal forest.

Figure 5 shows the relative runtimes of the RF‐based methods when using the CLM as the reference method (similar to Buczak et al., 2024). Relative runtimes have the advantage of being less dependent on the machine used for computation. As the CLM is the computationally least expensive method from the methods considered here, it serves as a sensible reference. For better visibility, the relative runtimes were logarithmized with a base of 10. As such, values of 0 indicate that a given method required the same runtime as the CLM in a given run, while a value of 1 indicates that a given method's runtime was larger than the CLM runtime by a factor of 10. It can be seen that from the set of RF‐based methods, fabOF achieved the lowest runtimes together with RF and naive OF. As RFSp had to fit four RF models in this case, it slightly trailed the three leading methods, as was to be expected. As already seen in Buczak et al. (2024), the optimization procedures make OF and OSOA computationally quite expensive. However, their runtime is also directly affected by the resources allotted to the optimization process (i.e., number of score sets for OF or maximum number of evaluations for OSOA). For OF and OSOA, changing the prediction procedure did not notably impact the runtime. This is not surprising as the bulk of the runtime is spent during the optimization procedure and the prediction method is only used once at the very end with the final model.

While fabOF achieved even lower runtimes than RF and naive OF, the differences between these three methods should not be overstated since the prototype fabOF implementation used for the simulation did not use any form of input checking which might add more overhead. Still, the results show a significant disparity in (relative) runtime between fabOF and OF which both were the best performing methods (using AFTA prediction for OF) when considering predictive performance. Since fabOF only needed to fit a single RF model and achieved results similar to those of OF with its extensive optimization procedure, this represents a meaningful advantage of fabOF.

## 5 SIMULATION STUDY 2: VARIABLE IMPORTANCE

Apart from evaluating the predictive performance of fabOF, I also evaluated the custom permutation VIM of fabOF using simulation data. To investigate whether the VIM consistently discovered the influential covariates, I computed the variable importance for data generated according to DGP 1 (including only normally distributed covariates) and DGP 4 (including 13 normally distributed and two binary covariates) from the first simulation study. For each DGP, I simulated 100 datasets of size 1000 and computed the variable importance using 100 permutation replications for a fabOF model consisting of 500 trees.

**FIGURE 6:**
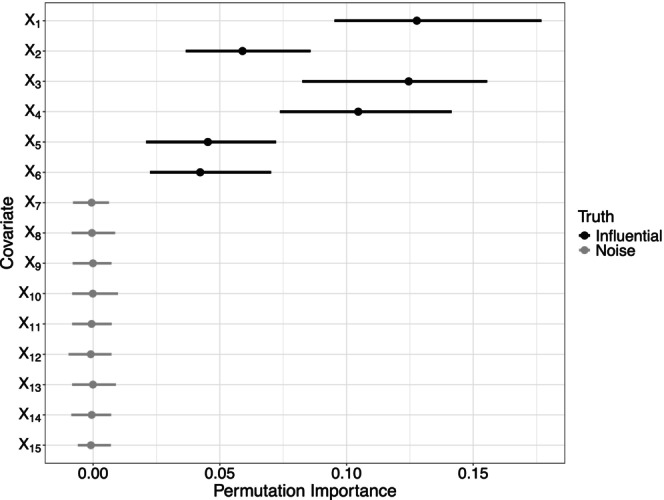
Permutation variable importance values for data generated using DGP 1. Colour coding indicates whether covariates were simulated as influential or as noise.

To visualize the results, I followed the iml package (Molnar et al., 2018), i.e., for each covariate the range of importance values between the 5% and 95% quantiles are displayed with a dot indicating the median variable importance. For DGP 1, Figure 6 shows that fabOF's VIM was able to recover the influential covariates quite well. All six influential covariates $X_{1},\text{…},X_{6}$ achieved notably higher importance values than the noise variables $X_{7},\text{…},X_{15}$. For the latter, importance values around 0 were mostly observed.

**FIGURE 7:**
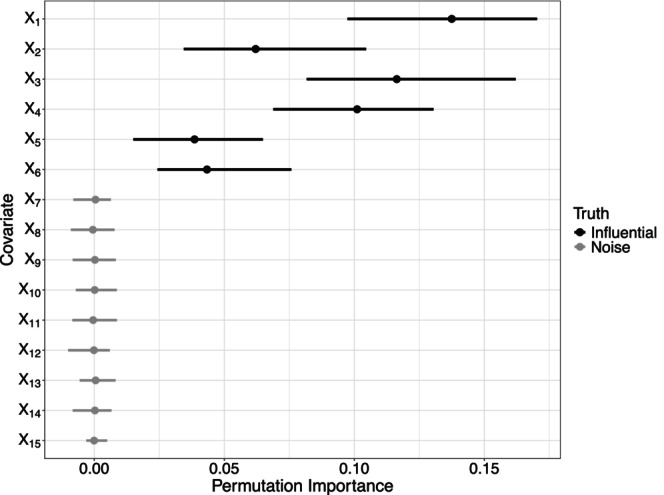
Permutation variable importance values for data generated using DGP 4. Colour coding indicates whether covariates were simulated as influential or as noise.

For DGP 4 in which $X_{5}$ and $X_{15}$ were replaced by binary covariates, a similar picture emerged in Figure 7. The permutation VIM was able to distinguish between influential and noise covariates fairly well. Again, the noise covariates achieved importance values around 0 while all influential covariates reached higher importance values. When comparing the importance of $X_{5}$ for DGP 1 (where it was a normally distributed covariate) and DGP 4 (where it was a binary covariate), it can be seen that the importance values were slightly lower for the binary case. A potential explanation could be that the effect magnitude was not translated equivalently when changing from to DGP 1 to DGP 4. Another explanation may be that RFs are known to be biased towards covariates with many different split points, which in turn can also affect VIM results (Strobl et al., 2007).

## 6 ILLUSTRATIVE DATA EXAMPLE

Apart from simulation data, I also evaluated fabOF on the basis of an illustrative data example on student performance in a language course from two Portuguese high schools. The original data were first introduced in Cortez and Silva (2008). I used the dataset provided in Cortez (2014) for this work, which consisted of 649 observations and 30 covariates with no missing values present. Further, I considered the same subset of the data already analysed in Buczak et al. (2024), which used 12 of the 30 original covariates. These included age, gender, residence (rural or urban), parental education status, parental cohabitation status, educational support from the family and the school, taking of private tutoring, internet access at home, and interest in pursuing higher education. The target variable is the final grade in a Portuguese language course. The original grades ranging from 0 to 20 were binned using the same binning values as in Cortez and Silva (2008), resulting in five categories: 0–9 (n=100), 10–11 (n=201), 12–13 (n=154), 14–15 (n=112), 16–20 (n=82). For comparison, I used the same prediction methods as in the first simulation study. Predictive performance was assessed using Cohen's weighted Kappa (Cohen, 1968) with linear and quadratic weights as well as Kendall's rank correlation (Kendall, 1945). To avoid overconfident performance values, I used a five‐fold cross‐validation (CV) with 50 replications, where the ordinal prediction models were trained on the respective training set of the CV partition and evaluated on the test set.

**FIGURE 8:**
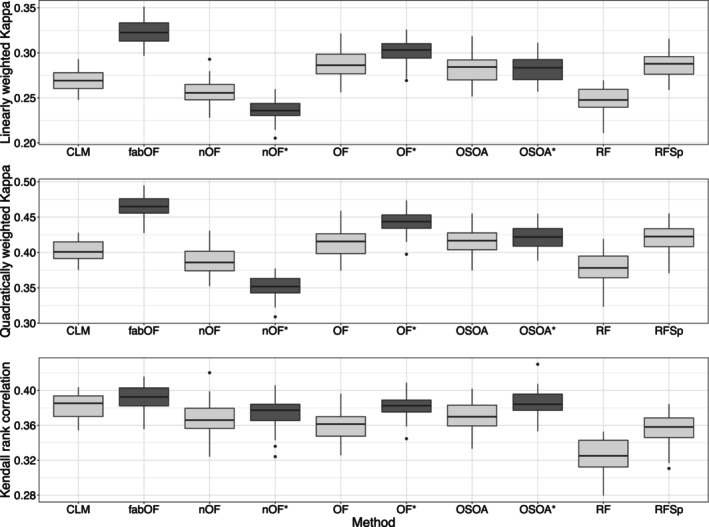
Predictive performance of existing methods and modifications for student performance data. Approaches using aggregate‐first‐transform‐after prediction indicated through dark grey coloured boxplots with additional asterisk indicating modification of existing method. CLM, cumulative link model (proportional odds); fabOF, Frequency‐Adjusted Borders Ordinal Forest; nOF, naive ordinal forest; OF, ordinal forest; OSOA, ordinal score optimization algorithm; RF, random forest; RFSp, split‐based ordinal forest.

Figure 8 shows that fabOF achieved the highest weighted Kappa values for both linear and quadratic weights, outperforming existing and AFTA‐modified methods. For Kendall's rank correlation, fabOF was slightly ahead as well, although the methods were closer in performance for this performance measure. For OF, AFTA prediction improved the predictive performance for all three measures. In the case of OSOA, performance increased slightly for Kendall's rank correlation but remained mostly unaffected for weighted Kappa. For naive OF, AFTA prediction slightly increased performance for Kendall's rank correlation but notably decreased it for Cohen's weighted Kappa.

Overall, the increased predictive performance of fabOF over existing methods is a promising finding as data‐driven prediction of student performance can play a key role in informing policymaking and the establishment of student support systems (Costa‐Mendes et al., 2020; van der Scheer & Visscher, 2017).

**FIGURE 9:**
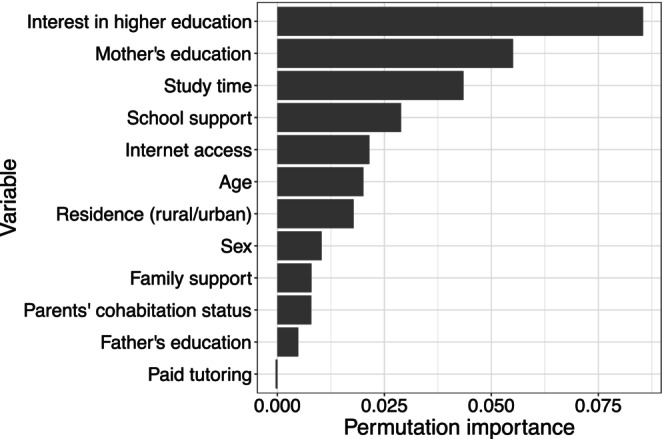
Permutation variable importance for student performance data.

For more detailed insights about the impact of individual covariates on the prediction of student performance, I computed variable importance values using fabOF's custom permutation VIM (with 100 replications). Figure 9 shows that the most important variables are an interest in higher education, mother's education, and study time. These findings are largely consistent with the educational research literature where interest in (higher) education has been found to be an important motivational variable associated with student performance (Hidi & Harackiewicz, 2000). Parental education has also been found to be a significant predictor of student achievement (e.g., Wößmann, 2003), with mothers particularly impacting a child's educational attainment (Cabus & Ariës, 2016). The low importance of father's education could potentially be partly explained by the relatively high correlation between mother's and father's education (Kendall's $\tau_{B}=0.57$). Including highly correlated variables can lead to importance being split between them (Molnar, 2022). When removing mother's education from the model, the importance of father's education increases comparatively (see Supporting Information). Research about the effect of study time on student achievement has been rather inconclusive, with some studies finding positive effects but others studies only finding an effect when the relation was mediated by motivational factors (see e.g., Keith, 1982; Masui et al., 2012; Rosário et al., 2012).

Overall, however, the presence of high correlations among some covariates indicates that the results should be interpreted with caution. VIMs are known to be affected by high correlations between covariates (Strobl et al., 2008). As a remedy, Strobl et al. (2008) proposed conditional permutation VIMs, which aim to preserve the original correlation structure in the data by restricting the way permutations can be performed. Therefore, comparing the foregoing results to variable importance results from a conditional VIM would be helpful to assess how reliable the interpretations are.

## 7 DISCUSSION

In this work, I proposed fabOF, a novel method for ordinal prediction that adds to the methodological stream of ordinal prediction methods based on RF (Breiman, 2001), such as ordinal forest (OF; Hornung, 2019), split‐based ordinal random forest (RFSp; Tutz, 2021), and the ordinal score optimization algorithm (OSOA; Buczak et al., 2024). Through simulation and an illustrative data example of student performance in a Portuguese language course (Cortez & Silva, 2008), I demonstrated that fabOF shows promising predictive performance and can improve upon existing methods in many data scenarios. Similar to OF and OSOA, fabOF assigns numeric scores to ordinal response categories and fits a regression RF using numeric scores as the target variable. For unseen observations, the predicted numeric scores from the RF fit are transformed into ordinal response categories using category borders that reflect a partition of the assumed latent variable's domain. Whereas in OF and OSOA numeric scores and category borders are directly linked (numeric scores are always chosen as the midpoint of the respective category interval as defined by the category borders), fabOF separates the choice of scores and category borders. Using default scores (i.e., 1,2,…,k for k categories), fabOF employs a heuristic for deriving adaptive category borders based on the cumulative relative frequencies of the response categories in the data. A simulation study showed that this approach was particularly effective in scenarios where response categories are not equally occupied. Furthermore, in comparison to OF and OSOA, the heuristic eliminates the need for an extensive optimization procedure in fabOF.

Buczak et al. (2024) found that in real data applications, differences in comparative predictive performance emerged, stressing the importance of evaluating ordinal prediction methods on various datasets. Apart from the student performance data covered in detail in this work, I have also benchmarked fabOF on seven other datasets used in Buczak et al. (2024). These additional results are included in the Supporting Information. For a detailed description of the datasets, see Buczak et al. (2024). Compared to the RF‐based ordinal prediction methods, fabOF achieved the highest predictive performance or places among the best performing methods for the majority of datasets. fabOF only fell behind notably for one dataset. Further investigation would be needed to determine whether one could infer more detailed reasons as to why fabOF did not perform well for this dataset or whether the data rather represented an outlier. Overall, however, these additional benchmarks underline the promising findings obtained from the simulation study and the student performance data presented here. Future work could also explore fabOF's performance in other data scenarios such as data generated from partial proportional odds models (see e.g., Brant, 1990; Peterson & Harrell, 1990), where not all covariate effects are global but some may instead differ across ordinal response categories.

Additionally, my simulation results indicate that the newly introduced prediction scheme of aggregating numeric score predictions at the tree level first and then transforming them to an ordinal response category via category borders (referred to as AFTA) as opposed to the reverse order (referred to as TFAA), which is currently employed in OF and OSOA, can yield benefits for OF and OSOA as well. Particularly for OF, AFTA prediction could improve predictive performance across all measures. For the simulation data, AFTA‐modified OF often reached similar performance values compared to fabOF. As both share the underlying regression RF framework, the same prediction scheme (AFTA) and both aim to find a high‐performing choice of category borders (through optimization or the heuristic), similar performance of these two approaches is likely commonly encountered. However, the simulation data and the real data example have also shown that the additional flexibility of fabOF (category borders and scores are decoupled from one another) and its frequency‐based heuristic can lead to performance advantages depending on the data at hand. However, this does not imply that fabOF will always perform at least as well as AFTA‐modified OF as such statements are generally not tenable in machine learning.

To enhance the interpretability of fabOF, I additionally introduced a custom permutation VIM based on Cohen's weighted Kappa (Cohen, 1968). Using simulated data, the permutation VIM was able to recover the influential covariates quite well. The importance of noise covariates was mostly around 0, while the importance of influential covariates was notably higher. When applied to the illustrative data example, findings that were largely consistent with the educational research literature emerged. However, it is not clear how fabOF's permutation VIM reacts to highly correlated covariates as the simulation data only included covariates which were simulated as uncorrelated. Unconditional VIMs (i.e., VIMs which do not restrict the permutation process in any way) can be affected by highly correlated covariates (Strobl et al., 2008). For the illustrative data example, high correlations between covariates were present. This should be taken into account when interpreting results. Future work could further evaluate fabOF's VIM in the presence of high correlations and consider the development of a conditional permutation VIM, as proposed by Strobl et al. (2008). To preserve the original correlation structure of the data, conditional permutation VIMs only permute covariates within certain regions of the covariate space. Future work could also study how the number of replications affects the stability of fabOF's VIM such that sensible compromises between stability and computation time can be derived.

Regarding methodological improvement of fabOF, future research could investigate further approaches to selecting appropriate category borders. For example, one could try to optimize category borders in a way similar to the optimization procedure in OSOA (while keeping the default numeric scores). In principle, one could also optimize numeric scores and category borders at the same time. However, this may pose a complex optimization problem. A possible remedy for this could be to employ a procedure in the spirit of the EM algorithm (Dempster et al., 1977), where the optimization procedure iterates between optimizing one while keeping the other fixed.

A further avenue for future research may be motivated by the student performance data used in this work. Typically, data from an educational context possess a hierarchical structure where individual observations are nested within groups, e.g., school classes. Another classic example of hierarchical data are longitudinal studies where multiple assessments of each person are performed, so individual assessments are nested within the respective individuals. Hierarchical data structures can introduce group‐specific effects that need to be accounted for in the modelling process, e.g., through the inclusion of additional group‐specific random effects as in the (generalized) linear mixed model literature (see e.g., Hedeker & Gibbons, 1994; Tutz & Hennevogl, 1996, for extensions to ordinal regression). To extend fabOF to hierarchical data, one could adapt an approach similar to that of Hajjem et al. (2011) or Sela and Simonoff (2012), as was used in multiple extensions of RF to hierarchical data for different response types (for an overview, see Hu & Szymczak, 2023). Since ordinal data from psychological fields are often characterized by a hierarchical structure, such an extension could be a promising endeavour for future work.

## AUTHOR CONTRIBUTIONS

**Philip Buczak:** conceptualization; methodology; software; writing – original draft; visualization; formal analysis; investigation; writing – review and editing; project administration.

## Supporting information


Supinfo S1.

## Data Availability

The R code for this work can be obtained from the corresponding OSF repository https://osf.io/fn8bg/. The accompanying R package fabOF can be obtained from https://github.com/phibuc/fabOF. The illustrative data example used were obtained from https://archive.ics.uci.edu/dataset/320/student+performance.

## APPENDIX A COMPUTATIONAL DETAILS

All computations were performed using R (R Core Team, 2022) version 4.2.1 with the packages ordinalForest (Hornung, 2022) for fitting (naive) OFs, ranger (Wright & Ziegler, 2017) for fitting multilabel classification RFs and ordinal (Christensen, 2022) for fitting proportional odds models with the clm() function. For OSOA I used the implementation provided by the authors in https://osf.io/v64d9/, while for RFSP I used the implementation provided by the author in https://github.com/GerhardTutz/ScoreFreeTrees. For all RF‐based methods, I used 500 trees, which is the default value for the RF implementations in the ranger (Wright & Ziegler, 2017) and randomForest (Liaw & Wiener, 2002) packages. For other hyperparameters I used the default values as provided in the respective implementations. In particular, I refrained from performing a hyperparameter tuning as I mainly compared RF‐based methods among themselves, and RFs have been shown to be relatively robust regarding their choice of hyperparameters (Probst et al., 2019). This design choice is in line with previous research from the field (e.g., Buczak et al., 2024; Hornung, 2019; Janitza et al., 2016; Tutz, 2021).

## APPENDIX B RESPONSE CATEGORY DISTRIBUTION PATTERNS AND THRESHOLD CHOICES

The two distribution patterns for DGPs 1, 3 and 4 from the simulation study were enforced through the choice of threshold values using the same approach as in Buczak et al. (2024). To this end, I determined threshold values for each DGP that for a simulated dataset of 100,000 observations approximately resulted in the targeted relative frequencies per category as displayed in Table B1.

**TABLE B1:** Targeted relative frequencies $\pi_{r},r=1,\text{…},k$ based on number of categories k and response category distribution pattern.

Categories	Pattern	Targeted relative frequencies						
		$\pi_{1}$	$\pi_{2}$	$\pi_{3}$	$\pi_{4}$	$\pi_{5}$	$\pi_{6}$	$\pi_{7}$
k=5	Equal	0.20	0.20	0.20	0.20	0.20	–	–
	Wide middle	0.11	0.22	0.33	0.22	0.11	–	–
k=7	Equal	0.14	0.14	0.14	0.14	0.14	0.14	0.14
	Wide middle	0.06	0.13	0.19	0.25	0.19	0.13	0.06

For DGP 2 in which data were simulated from a linear regression model and binned into ordinal categories, the distribution patterns were obtained using specific binning values. To determine suitable binning values, I followed the approach in Buczak et al. (2024) of approximating the empirical distribution function of the numeric outcome through 100,000 simulated observations. The quantiles matching the intended relative frequencies per category in Table B1 were selected as binning values. Table B2 displays the values chosen as thresholds for DGPs 1, 3, and 4 as well as the binning values for DGP 2.

**TABLE B2:** Threshold/binning values for combinations of DGP, number of categories k, and response category distribution pattern.

DGP	Categories	Pattern	Threshold/binning values						
			$\gamma_{1}$	$\gamma_{2}$	$\gamma_{3}$	$\gamma_{4}$	$\gamma_{5}$	$\gamma_{6}$	$\gamma_{7}$
DGP 1	k=5	Equal	−3.25	−1.75	−0.5	1	∞	–	–
		Wide middle	−4	−2.25	−0.25	1.75	∞	–	–
	k=7	Equal	−3.75	−2.5	−1.6	−0.75	0.2	1.4	∞
		Wide middle	−5	−3.25	−1.85	−0.35	1.1	2.65	∞
DGP 2	k=5	Equal	−0.5	0.7	1.7	2.8	∞	–	–
		Wide middle	−1.25	0.3	2	3.5	∞	–	–
	k=7	Equal	−0.95	0	0.8	1.5	2.2	3.1	∞
		Wide middle	−1.9	−0.5	0.6	1.8	3	4.4	∞
DGP 3	k=5	Equal	−2.5	−1.25	−0.25	1	∞	–	–
		Wide middle	−3.25	−1.5	0.25	2	∞	–	–
	k=7	Equal	−3	−2	−1.15	−0.4	0.4	1.5	∞
		Wide middle	−4	−2.5	−1.35	0	1.25	2.65	∞
DGP 4	k=5	Equal	−3.7	−2.3	−1.1	0.3	∞	–	–
		Wide middle	−4.65	−2.7	−0.6	1.3	∞	–	–
	k=7	Equal	−4.25	−3	−2.1	−1.25	−0.3	0.9	∞
		Wide middle	−5.4	−3.7	−2.35	−1	0.4	2	∞

## APPENDIX C COHEN'S WEIGHTED KAPPA

### C.1

Cohen's weighted Kappa (Cohen, 1968) $\kappa_{w}$ is given by

$$\kappa_{w}=\frac{\overset{k}{\underset{r=1}{\sum }}\overset{k}{\underset{s=1}{\sum }}w_{rs}p_{rs}^{o}-\overset{k}{\underset{r=1}{\sum }}\overset{k}{\underset{s=1}{\sum }}w_{rs}p_{rs}^{c}}{1-\overset{k}{\underset{r=1}{\sum }}\overset{k}{\underset{s=1}{\sum }}w_{rs}p_{rs}^{c}},$$

where $p_{rs}^{o}$ denotes the observed proportion of instances with true category r and predicted category s, and $p_{rs}^{c}$ denotes the proportion that is expected by chance (Cohen, 1968). Furthermore, $w_{rs}$ denotes the weight assigned to instances with true category r and predicted category s. Weights must be specified in advance and act as a way of penalization regarding the distance between the true and predicted response category. In this work, I have used linear and quadratic weights, given by

$$\begin{array}{ll} w_{rs}^{\text{lin}} & =1-\frac{\left|r-s\right|}{k-1},\;\\ w_{rs}^{\text{quad}} & =1-\frac{|r-s{|}^{2}}{{\left(k-1\right)}^{2}}\; \end{array}$$

as these are common choices for ordinal prediction (Ben‐David, 2008; Buczak et al., 2024; Hornung, 2019). Compared to quadratic weights, linear weights place higher weight on instances for which the predicted categories are close or equal to the true categories. On the other hand, quadratic weights assign more weight to instances with predicted categories further away from the true categories than linear weights (Hornung, 2019).
