# Supplementary material for: Frequency‐adjusted borders ordinal forest: A novel tree ensemble method for ordinal prediction
Source: Br J Math Stat Psychol. 2024 Dec 8;78(2):594–616. doi: 10.1111/bmsp.12375 (PMC11971599; doi:10.1111/bmsp.12375)
Supplement: Supplementary file 1 — Supinfo S1. [file BMSP-78-594-s001.pdf]

Frequency-Adjusted Borders Ordinal Forest: A Novel Tree Ensemble Method for Ordinal Prediction – Supplement

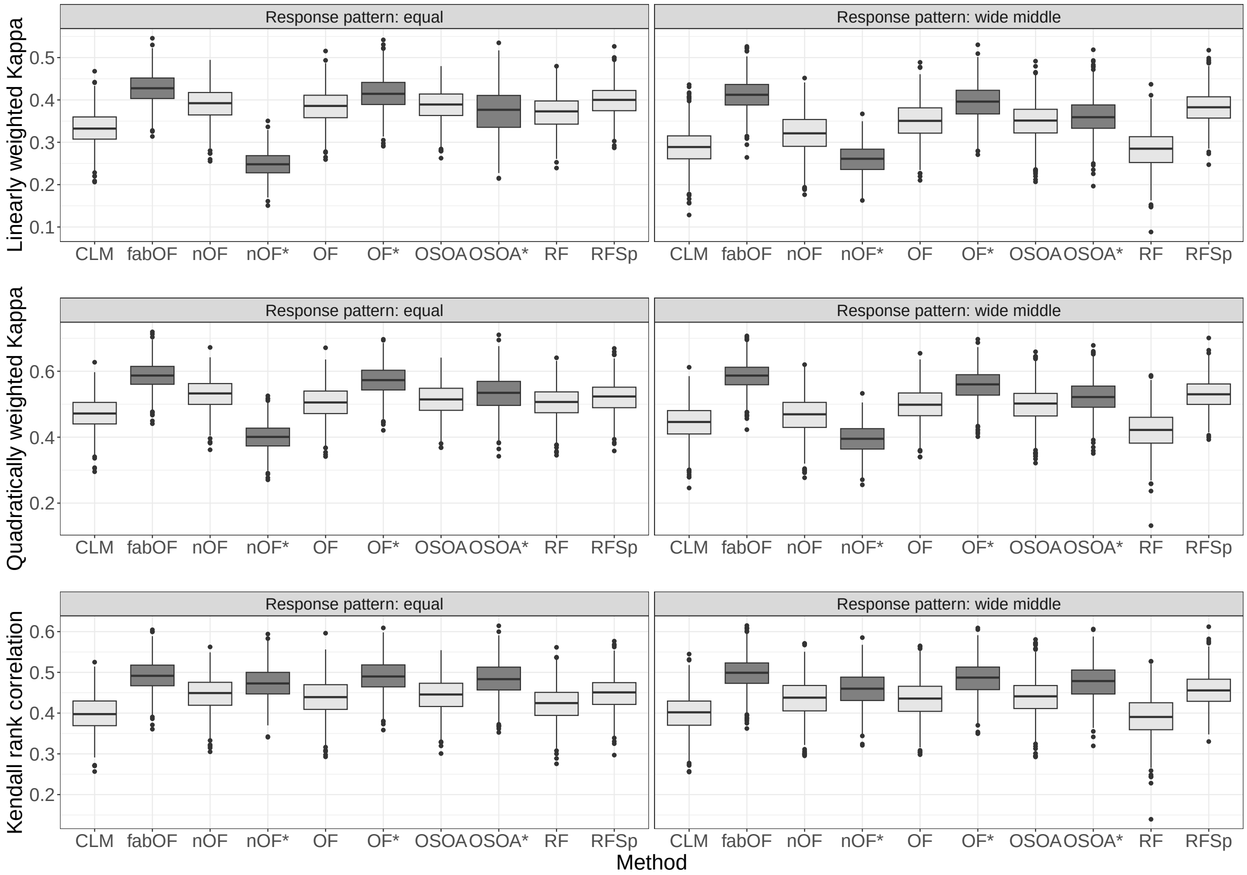

Figure 1

Predictive performance of all methods and modifications for data simulated from DGP 1 with $n = 750$, $k = 5$ and random sampling for both response category distribution patterns. Approaches using AFTA prediction indicated through dark grey colored boxplots with additional asterisk indicating modification of existing method.

Note: CLM: Cumulative Link Model (proportional odds), fabOF: Frequency-Adjusted Borders Ordinal Forest, nOF: naive Ordinal Forest, OF: Ordinal Forest, OSOA: Ordinal Score Optimization Algorithm, RF: Random Forest, RFSp: Split-based Ordinal Forest.

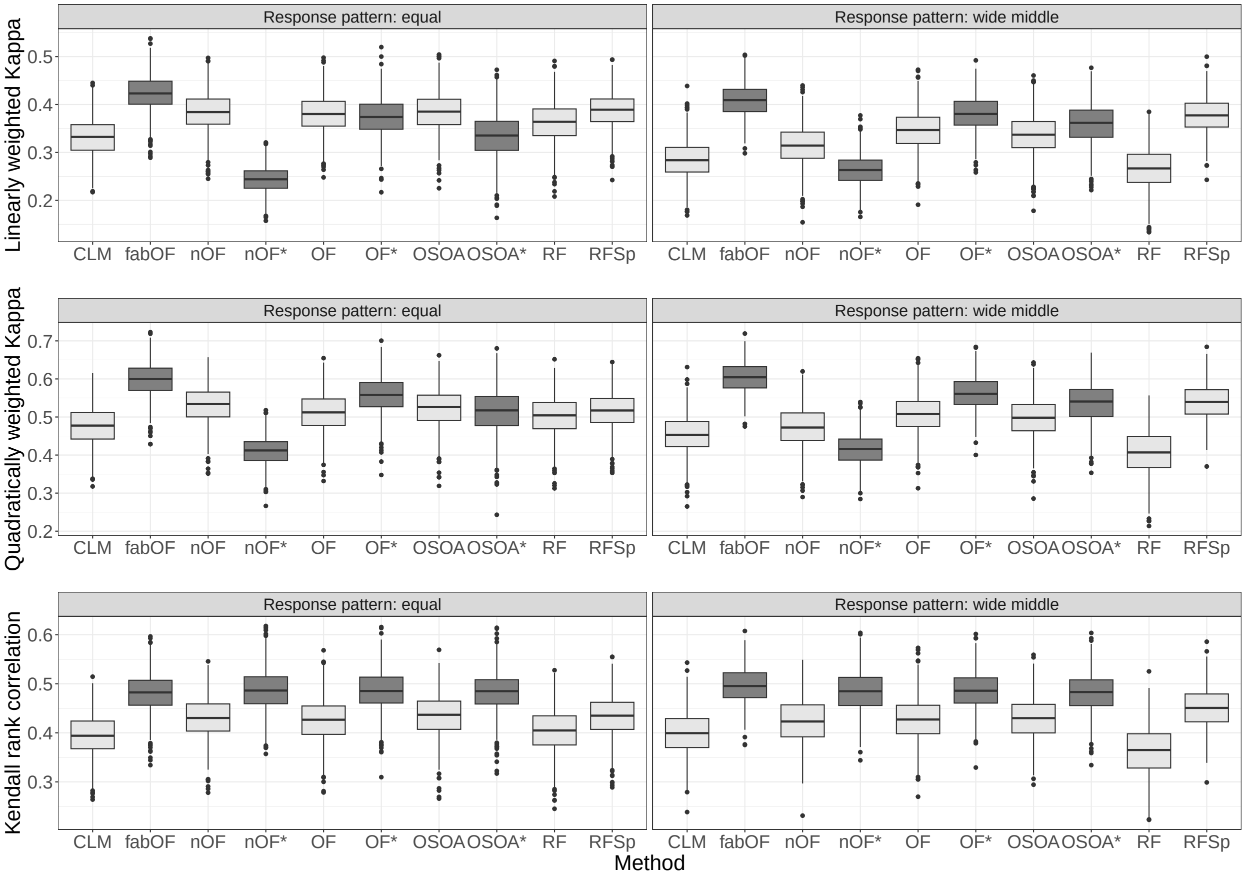

Figure 2

Predictive performance of all methods and modifications for data simulated from DGP 1 with $n = 750$, $k = 7$ and stratified sampling for both response category distribution patterns. Approaches using AFTA prediction indicated through dark grey colored boxplots with additional asterisk indicating modification of existing method.

Note: CLM: Cumulative Link Model (proportional odds), fabOF: Frequency-Adjusted Borders Ordinal Forest, nOF: naive Ordinal Forest, OF: Ordinal Forest, OSOA: Ordinal Score Optimization Algorithm, RF: Random Forest, RFSp: Split-based Ordinal Forest.

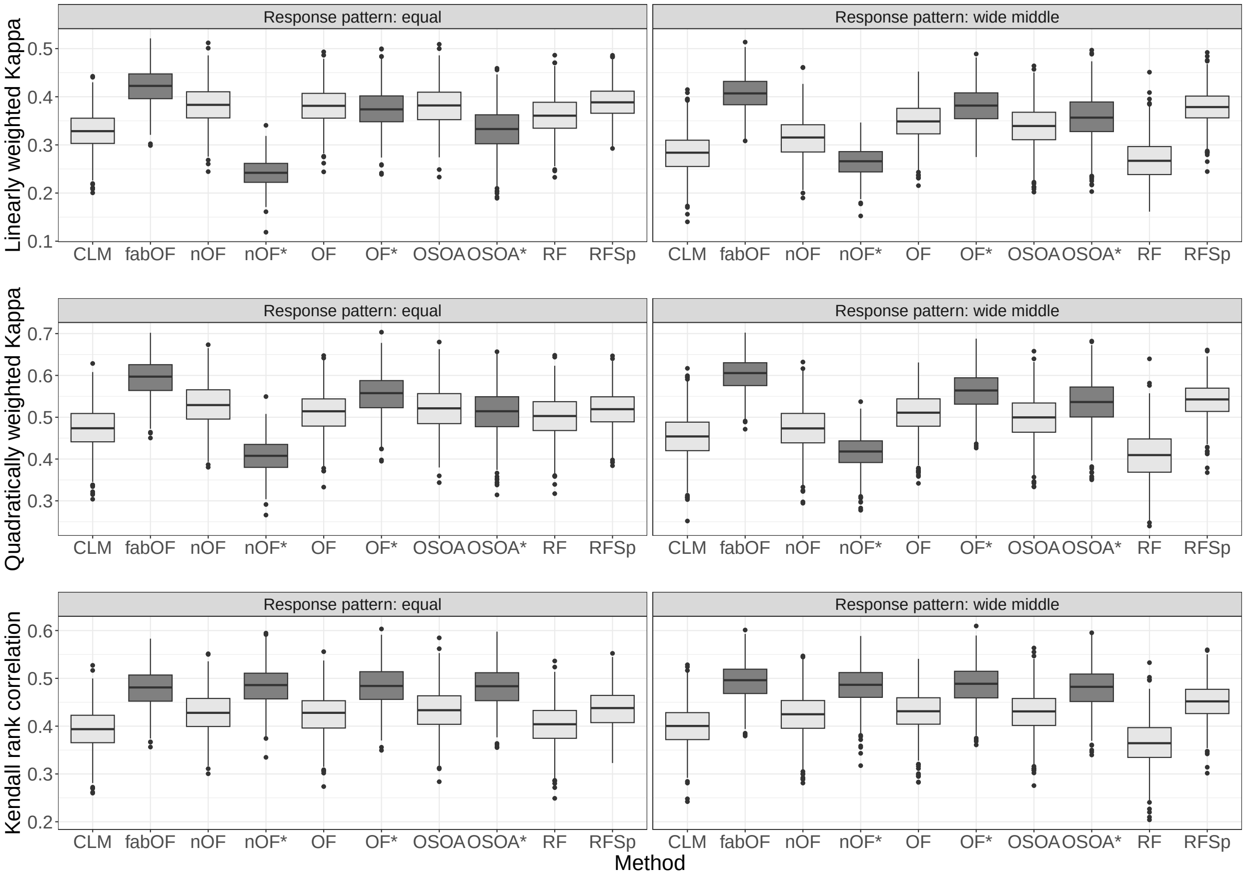

Figure 3

Predictive performance of all methods and modifications for data simulated from DGP 1 with $n = 750$, $k = 7$ and random sampling for both response category distribution patterns. Approaches using AFTA prediction indicated through dark grey colored boxplots with additional asterisk indicating modification of existing method.

Note: CLM: Cumulative Link Model (proportional odds), fabOF: Frequency-Adjusted Borders Ordinal Forest, nOF: naive Ordinal Forest, OF: Ordinal Forest, OSOA: Ordinal Score Optimization Algorithm, RF: Random Forest, RFSp: Split-based Ordinal Forest.

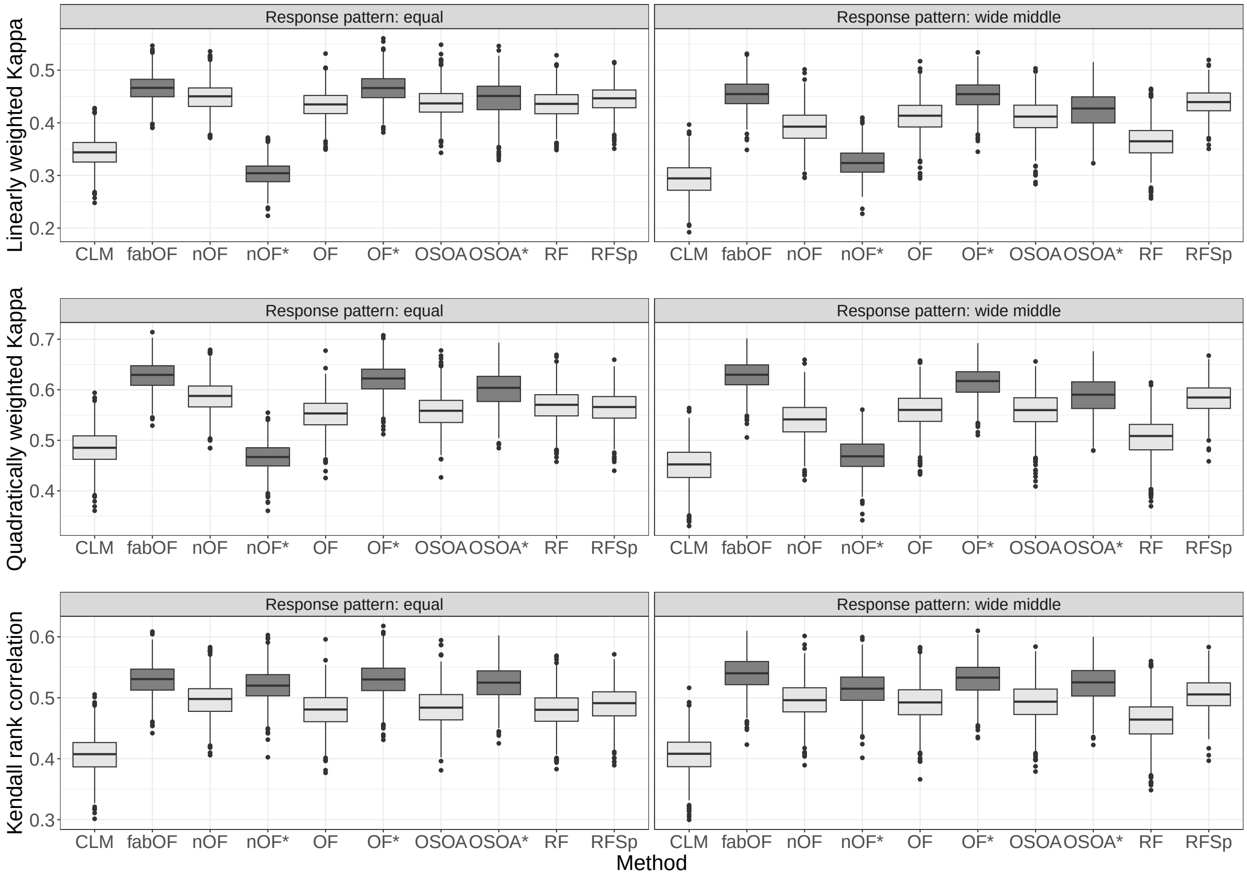

Figure 4

Predictive performance of all methods and modifications for data simulated from DGP 1 with $n = 1500$, $k = 5$ and stratified sampling for both response category distribution patterns. Approaches using AFTA prediction indicated through dark grey colored boxplots with additional asterisk indicating modification of existing method.

Note: CLM: Cumulative Link Model (proportional odds), fabOF: Frequency-Adjusted Borders Ordinal Forest, nOF: naive Ordinal Forest, OF: Ordinal Forest, OSOA: Ordinal Score Optimization Algorithm, RF: Random Forest, RFSp: Split-based Ordinal Forest.

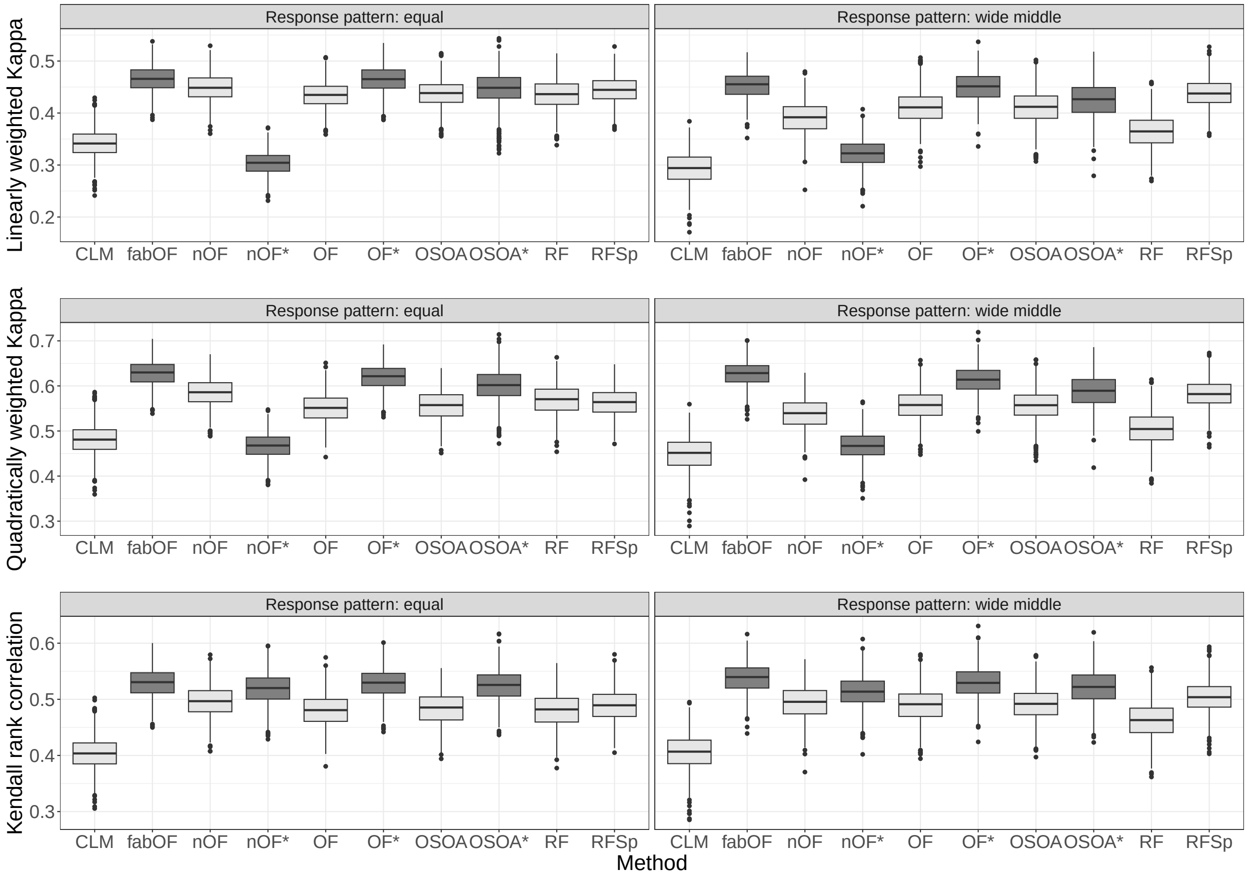

Figure 5

Predictive performance of all methods and modifications for data simulated from DGP 1 with $n = 1500$, $k = 5$ and random sampling for both response category distribution patterns. Approaches using AFTA prediction indicated through dark grey colored boxplots with additional asterisk indicating modification of existing method.

Note: CLM: Cumulative Link Model (proportional odds), fabOF: Frequency-Adjusted Borders Ordinal Forest, nOF: naive Ordinal Forest, OF: Ordinal Forest, OSOA: Ordinal Score Optimization Algorithm, RF: Random Forest, RFSp: Split-based Ordinal Forest.

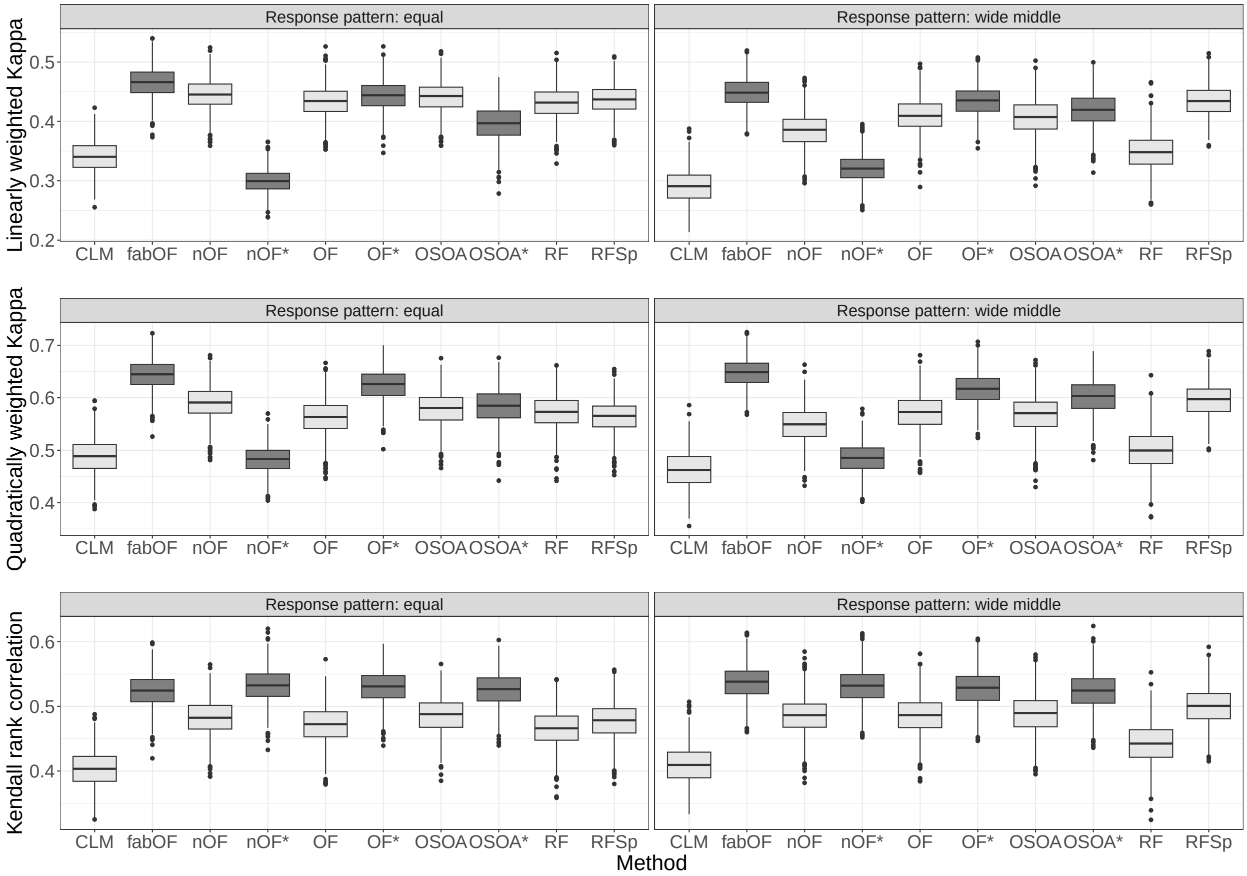

Figure 6

Predictive performance of all methods and modifications for data simulated from DGP 1 with $n = 1500$, $k = 7$ and stratified sampling for both response category distribution patterns. Approaches using AFTA prediction indicated through dark grey colored boxplots with additional asterisk indicating modification of existing method.

Note: CLM: Cumulative Link Model (proportional odds), fabOF: Frequency-Adjusted Borders Ordinal Forest, nOF: naive Ordinal Forest, OF: Ordinal Forest, OSOA: Ordinal Score Optimization Algorithm, RF: Random Forest, RFSp: Split-based Ordinal Forest.

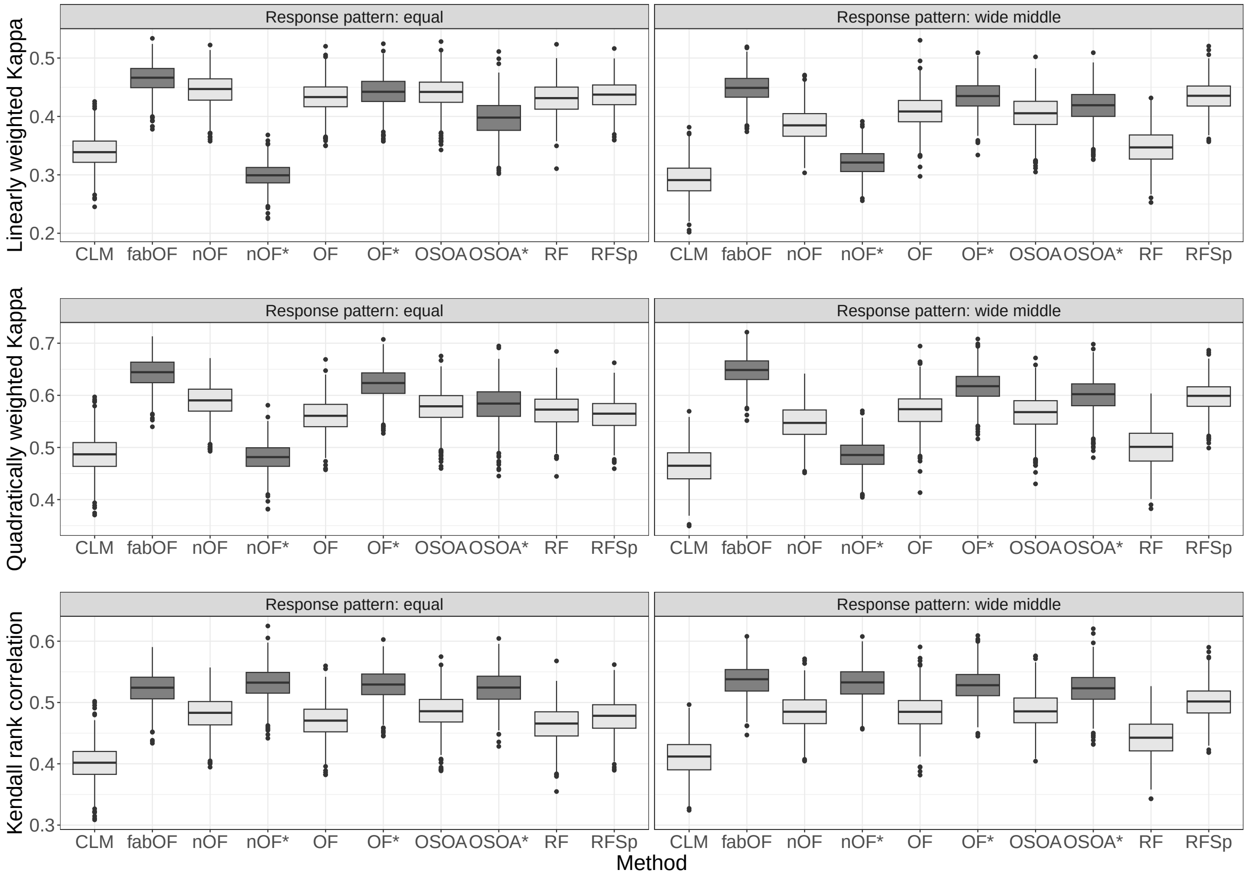

Figure 7

Predictive performance of all methods and modifications for data simulated from DGP 1 with $n = 1500$, $k = 7$ and random sampling for both response category distribution patterns. Approaches using AFTA prediction indicated through dark grey colored boxplots with additional asterisk indicating modification of existing method.

Note: CLM: Cumulative Link Model (proportional odds), fabOF: Frequency-Adjusted Borders Ordinal Forest, nOF: naive Ordinal Forest, OF: Ordinal Forest, OSOA: Ordinal Score Optimization Algorithm, RF: Random Forest, RFSp: Split-based Ordinal Forest.

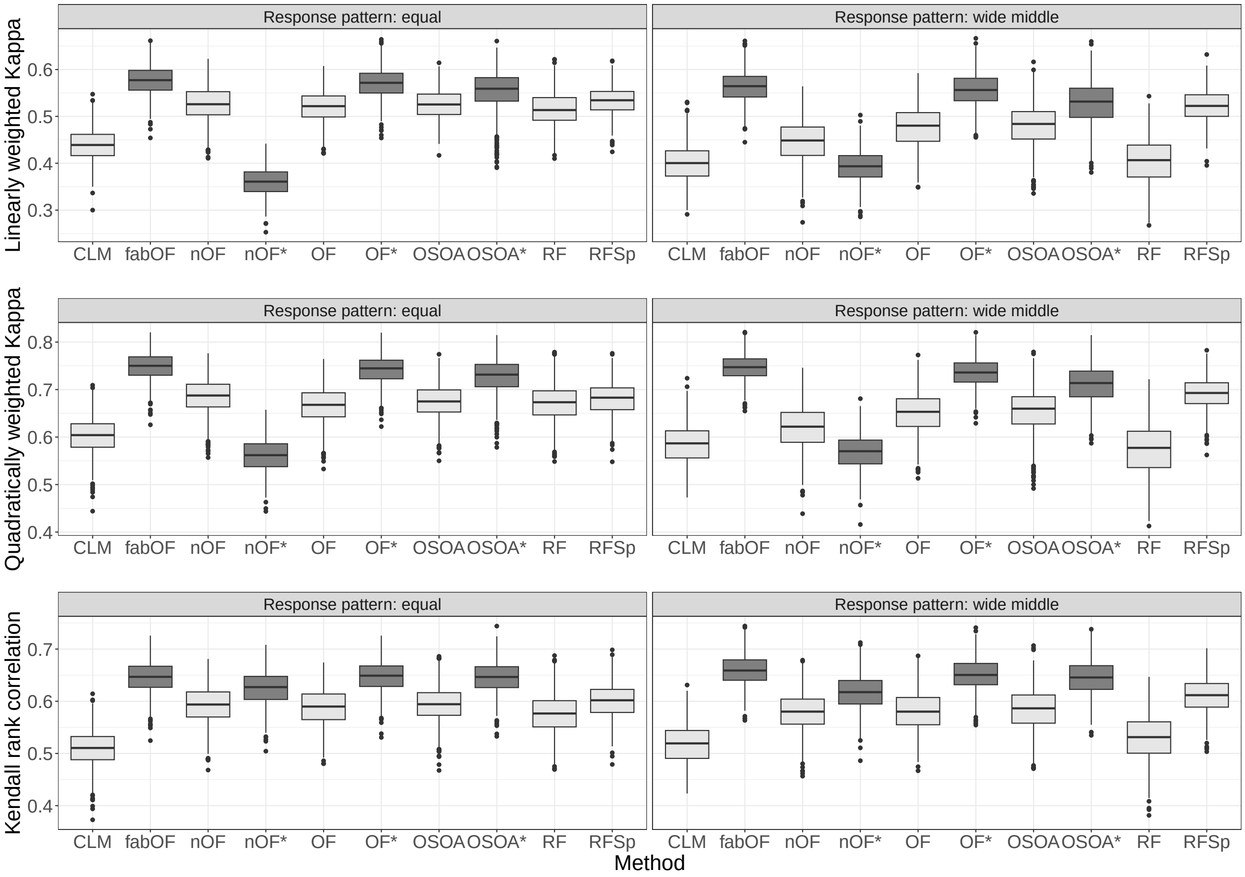

Figure 8

Predictive performance of all methods and modifications for data simulated from DGP 2 with $n = 750$, $k = 5$ and random sampling for both response category distribution patterns. Approaches using AFTA prediction indicated through dark grey colored boxplots with additional asterisk indicating modification of existing method.

Note: CLM: Cumulative Link Model (proportional odds), fabOF: Frequency-Adjusted Borders Ordinal Forest, nOF: naive Ordinal Forest, OF: Ordinal Forest, OSOA: Ordinal Score Optimization Algorithm, RF: Random Forest, RFSp: Split-based Ordinal Forest.

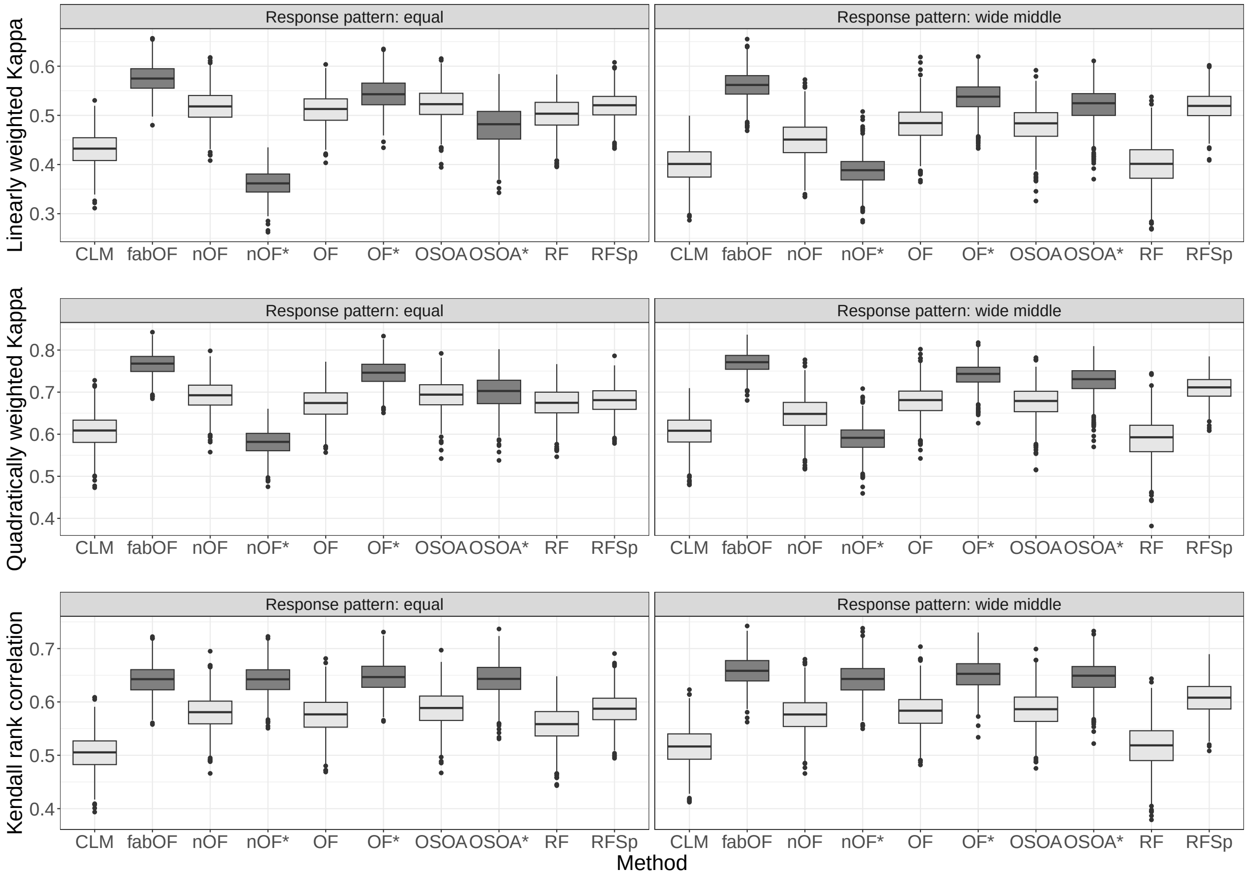

Figure 9

Predictive performance of all methods and modifications for data simulated from DGP 2 with $n = 750$, $k = 7$ and stratified sampling for both response category distribution patterns. Approaches using AFTA prediction indicated through dark grey colored boxplots with additional asterisk indicating modification of existing method.

Note: CLM: Cumulative Link Model (proportional odds), fabOF: Frequency-Adjusted Borders Ordinal Forest, nOF: naive Ordinal Forest, OF: Ordinal Forest, OSOA: Ordinal Score Optimization Algorithm, RF: Random Forest, RFSp: Split-based Ordinal Forest.

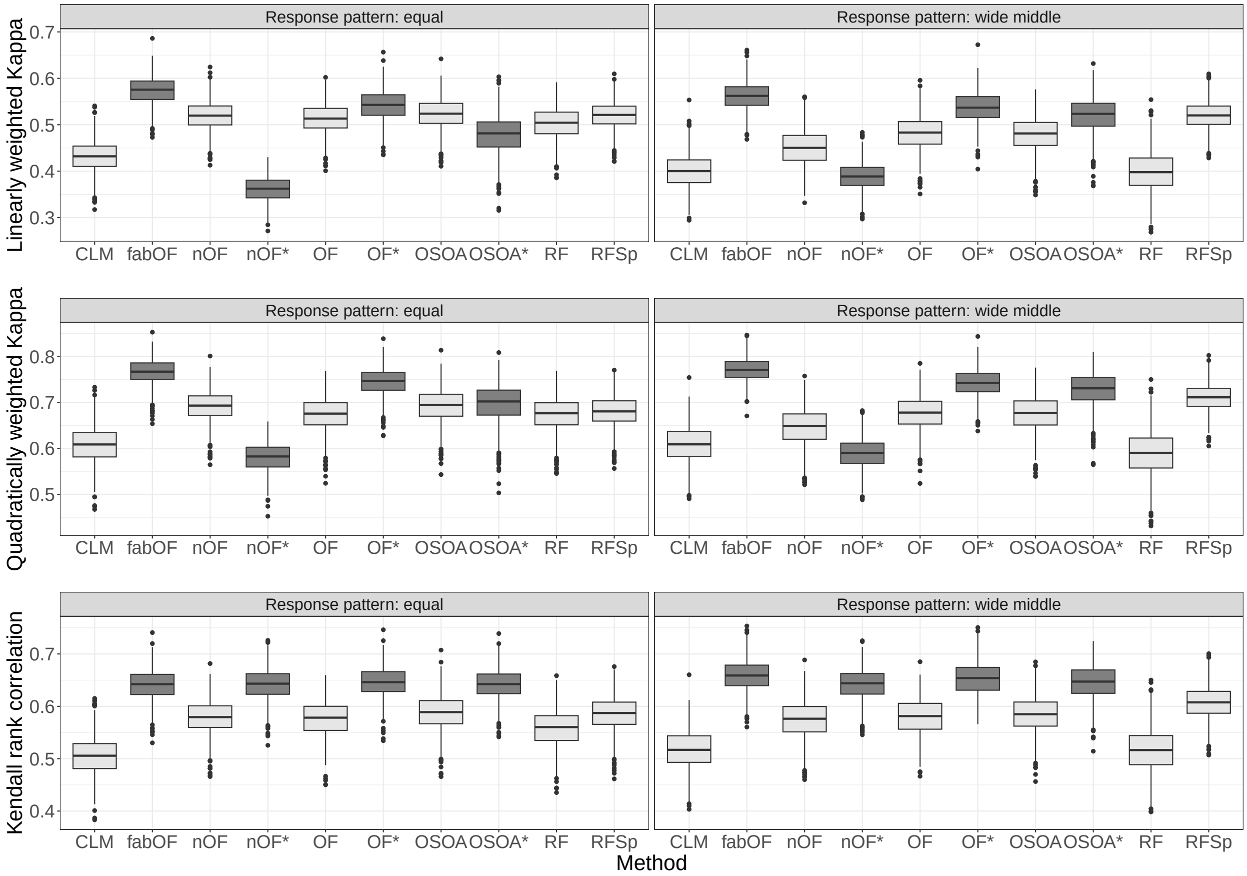

Figure 10

Predictive performance of all methods and modifications for data simulated from DGP 2 with $n = 750$, $k = 7$ and random sampling for both response category distribution patterns. Approaches using AFTA prediction indicated through dark grey colored boxplots with additional asterisk indicating modification of existing method.

Note: CLM: Cumulative Link Model (proportional odds), fabOF: Frequency-Adjusted Borders Ordinal Forest, nOF: naive Ordinal Forest, OF: Ordinal Forest, OSOA: Ordinal Score Optimization Algorithm, RF: Random Forest, RFSp: Split-based Ordinal Forest.

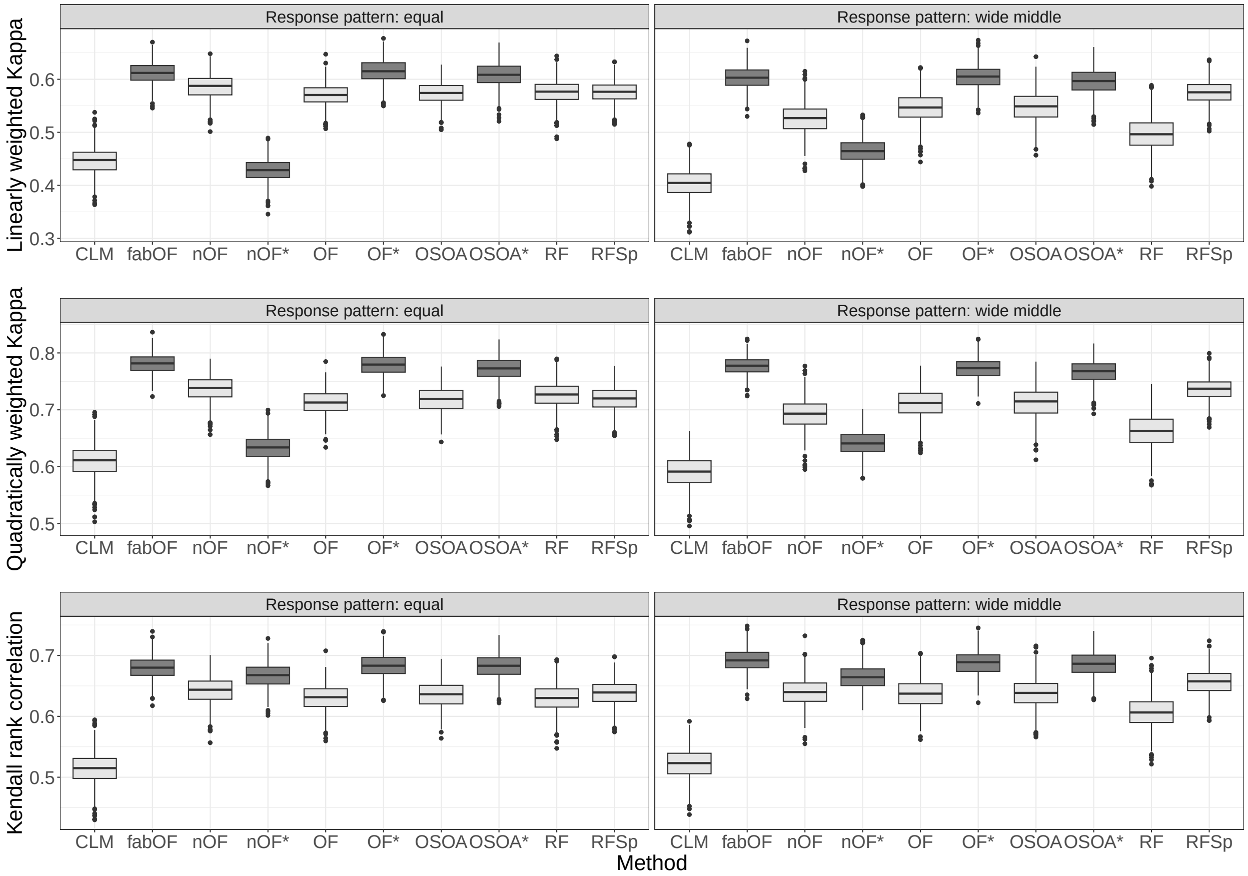

Figure 11

Predictive performance of all methods and modifications for data simulated from DGP 2 with $n = 1500$, $k = 5$ and stratified sampling for both response category distribution patterns. Approaches using AFTA prediction indicated through dark grey colored boxplots with additional asterisk indicating modification of existing method.

Note: CLM: Cumulative Link Model (proportional odds), fabOF: Frequency-Adjusted Borders Ordinal Forest, nOF: naive Ordinal Forest, OF: Ordinal Forest, OSOA: Ordinal Score Optimization Algorithm, RF: Random Forest, RFSp: Split-based Ordinal Forest.

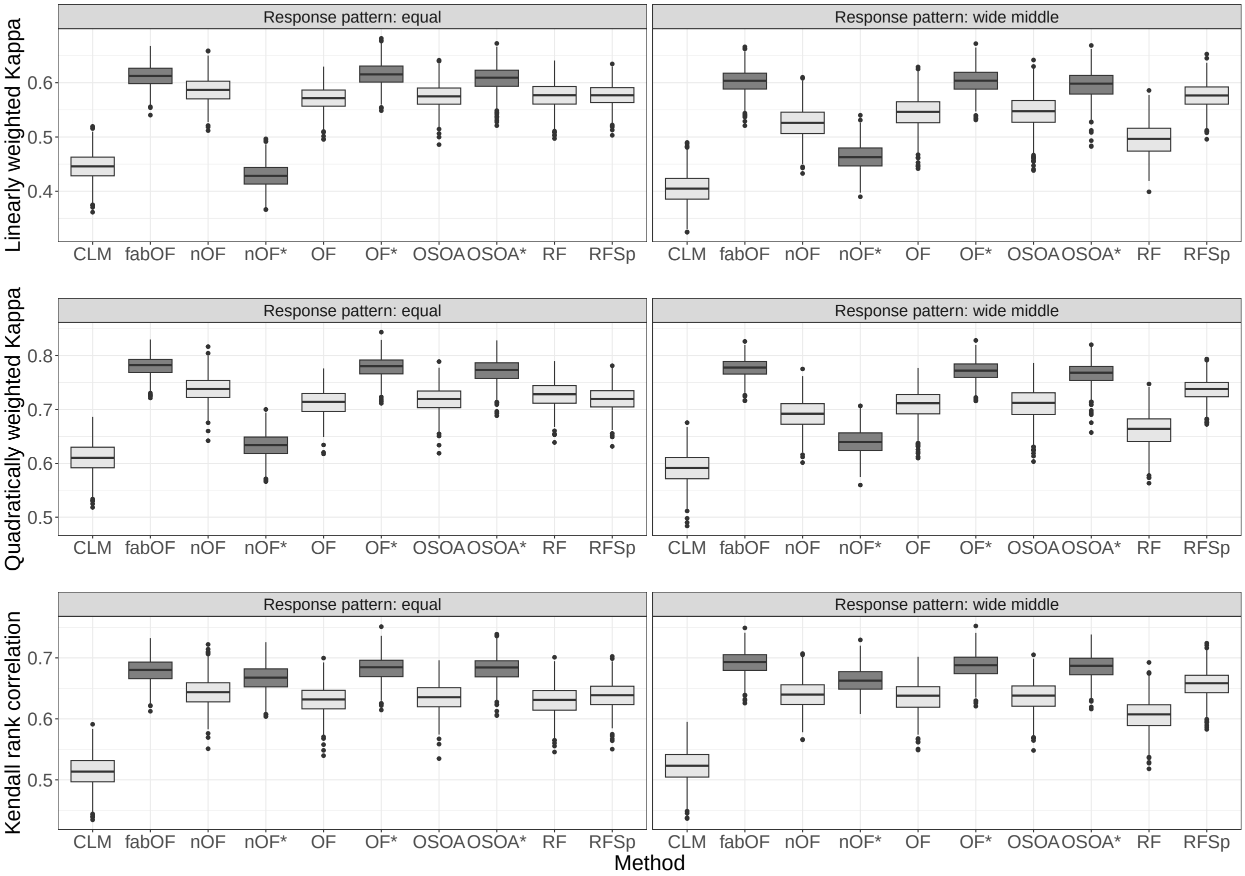

Figure 12

Predictive performance of all methods and modifications for data simulated from DGP 2 with $n = 1500$, $k = 5$ and random sampling for both response category distribution patterns. Approaches using AFTA prediction indicated through dark grey colored boxplots with additional asterisk indicating modification of existing method.

Note: CLM: Cumulative Link Model (proportional odds), fabOF: Frequency-Adjusted Borders Ordinal Forest, nOF: naive Ordinal Forest, OF: Ordinal Forest, OSOA: Ordinal Score Optimization Algorithm, RF: Random Forest, RFSp: Split-based Ordinal Forest.

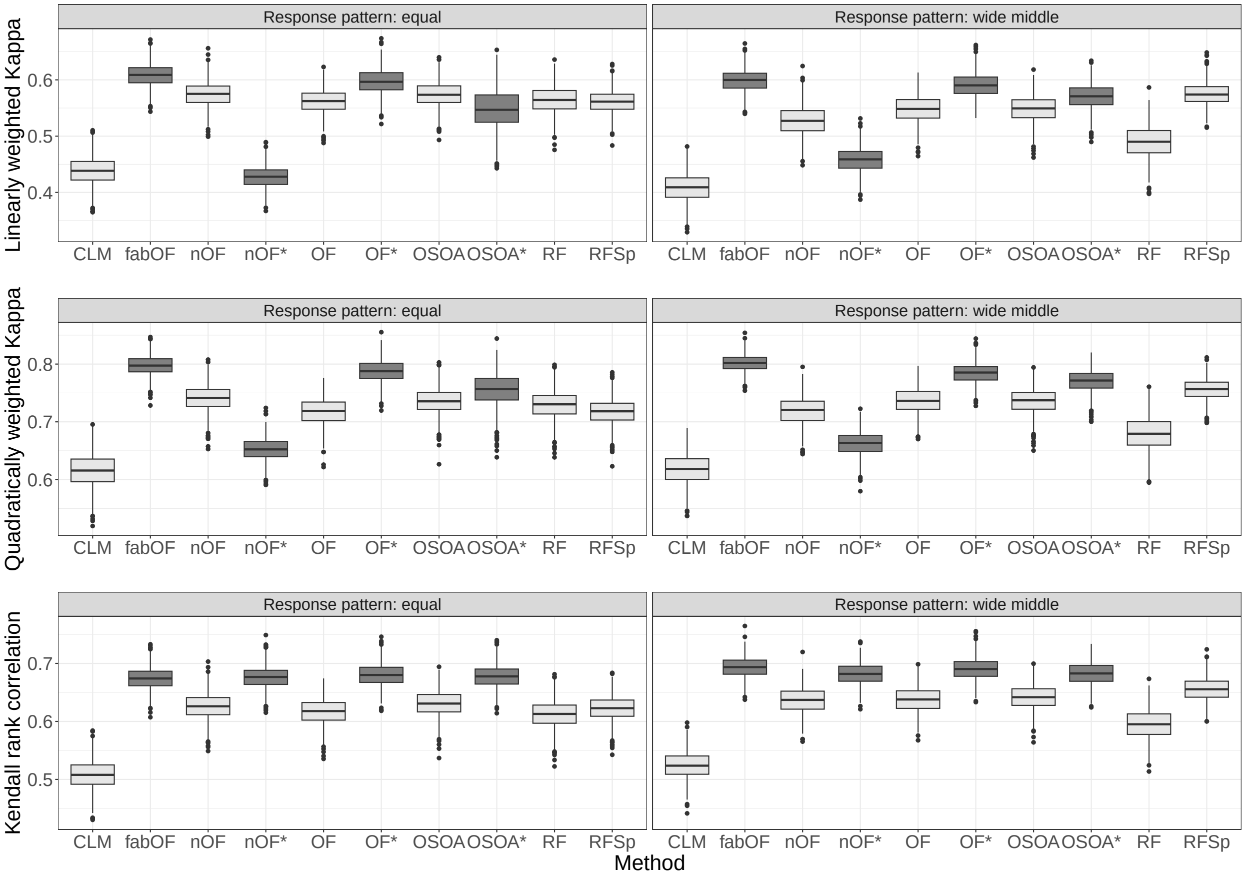

Figure 13

Predictive performance of all methods and modifications for data simulated from DGP 2 with $n = 1500$, $k = 7$ and stratified sampling for both response category distribution patterns. Approaches using AFTA prediction indicated through dark grey colored boxplots with additional asterisk indicating modification of existing method.

Note: CLM: Cumulative Link Model (proportional odds), fabOF: Frequency-Adjusted Borders Ordinal Forest, nOF: naive Ordinal Forest, OF: Ordinal Forest, OSOA: Ordinal Score Optimization Algorithm, RF: Random Forest, RFSp: Split-based Ordinal Forest.

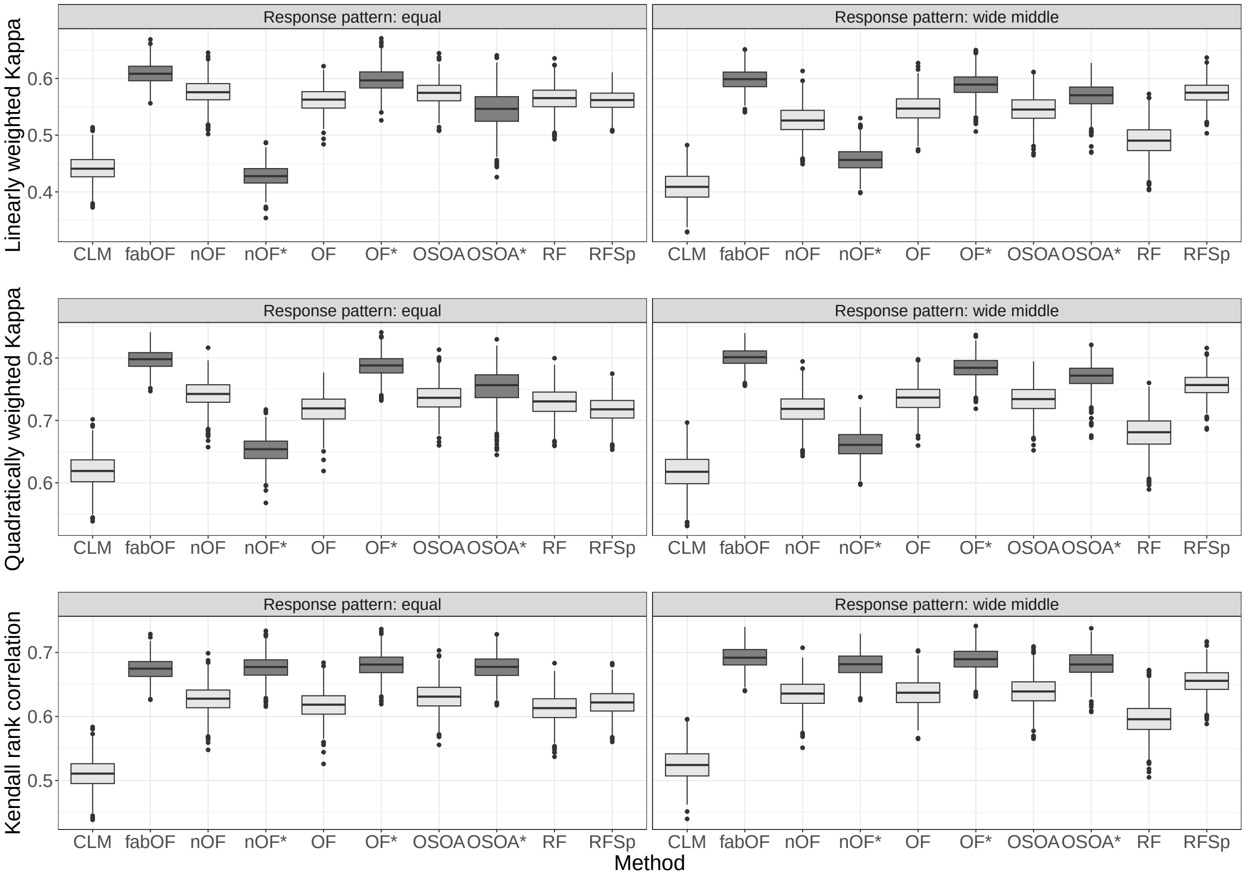

Figure 14

Predictive performance of all methods and modifications for data simulated from DGP 2 with $n = 1500$, $k = 7$ and random sampling for both response category distribution patterns. Approaches using AFTA prediction indicated through dark grey colored boxplots with additional asterisk indicating modification of existing method.

Note: CLM: Cumulative Link Model (proportional odds), fabOF: Frequency-Adjusted Borders Ordinal Forest, nOF: naive Ordinal Forest, OF: Ordinal Forest, OSOA: Ordinal Score Optimization Algorithm, RF: Random Forest, RFSp: Split-based Ordinal Forest.

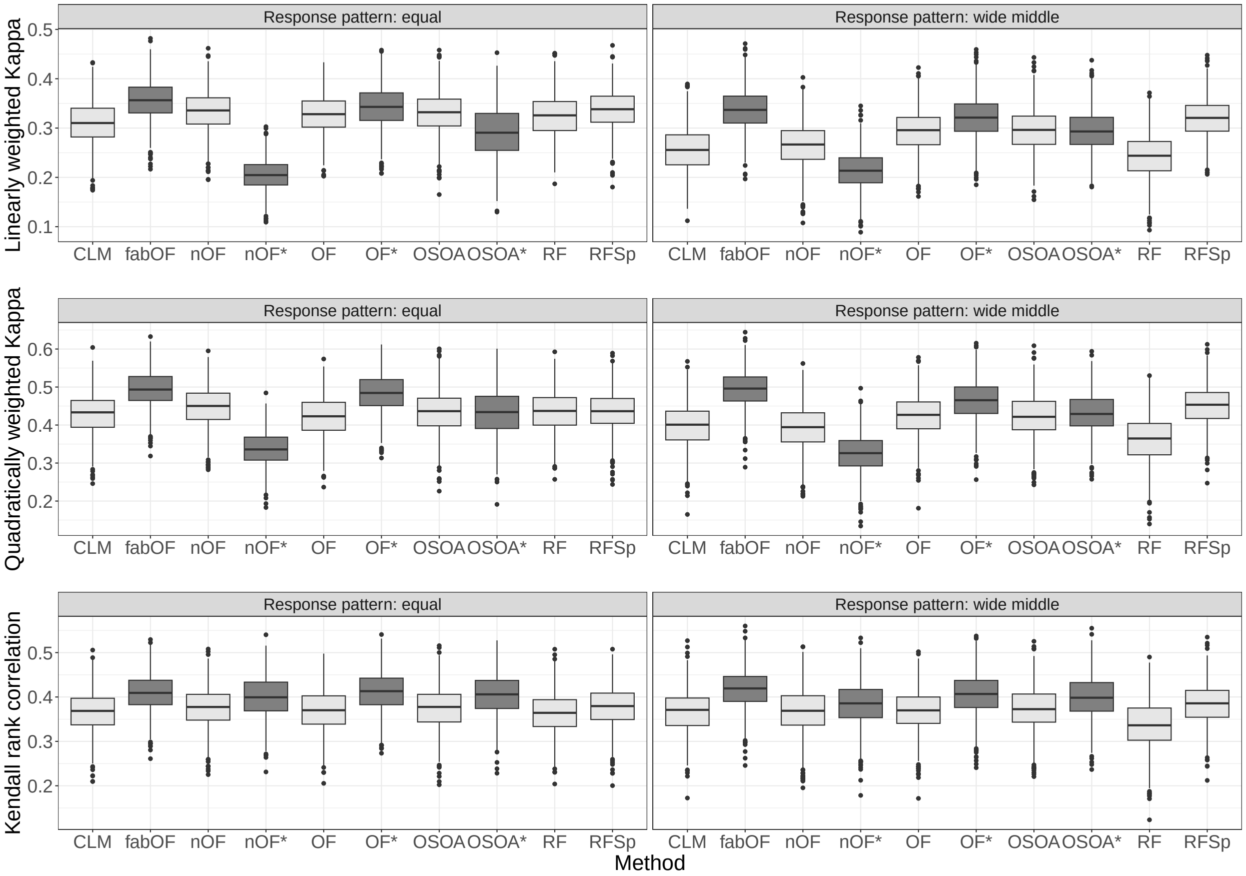

Figure 15

Predictive performance of all methods and modifications for data simulated from DGP 3 with $n = 750$, $k = 5$ and random sampling for both response category distribution patterns. Approaches using AFTA prediction indicated through dark grey colored boxplots with additional asterisk indicating modification of existing method.

Note: CLM: Cumulative Link Model (proportional odds), fabOF: Frequency-Adjusted Borders Ordinal Forest, nOF: naive Ordinal Forest, OF: Ordinal Forest, OSOA: Ordinal Score Optimization Algorithm, RF: Random Forest, RFSp: Split-based Ordinal Forest.

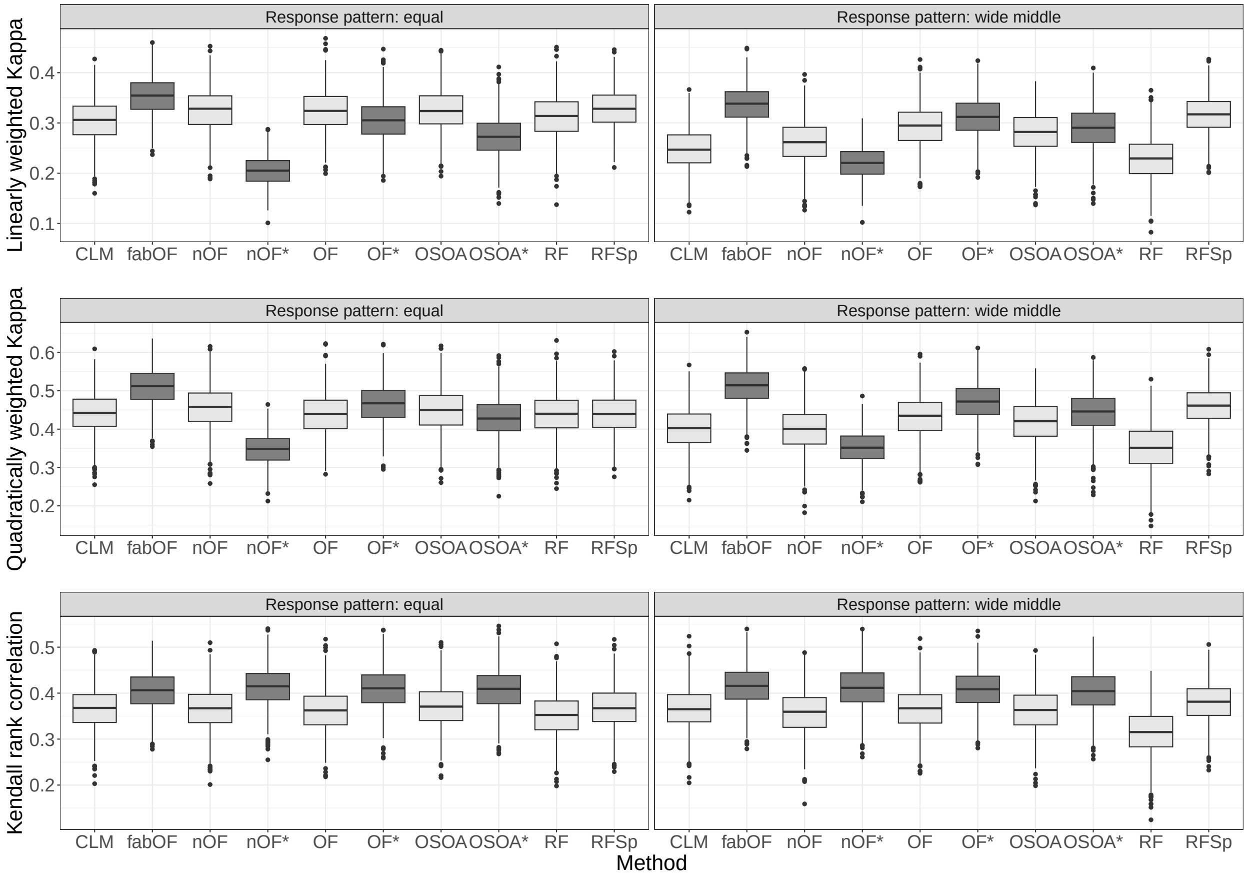

Figure 16

Predictive performance of all methods and modifications for data simulated from DGP 3 with $n = 750$, $k = 7$ and stratified sampling for both response category distribution patterns. Approaches using AFTA prediction indicated through dark grey colored boxplots with additional asterisk indicating modification of existing method.

Note: CLM: Cumulative Link Model (proportional odds), fabOF: Frequency-Adjusted Borders Ordinal Forest, nOF: naive Ordinal Forest, OF: Ordinal Forest, OSOA: Ordinal Score Optimization Algorithm, RF: Random Forest, RFSp: Split-based Ordinal Forest.

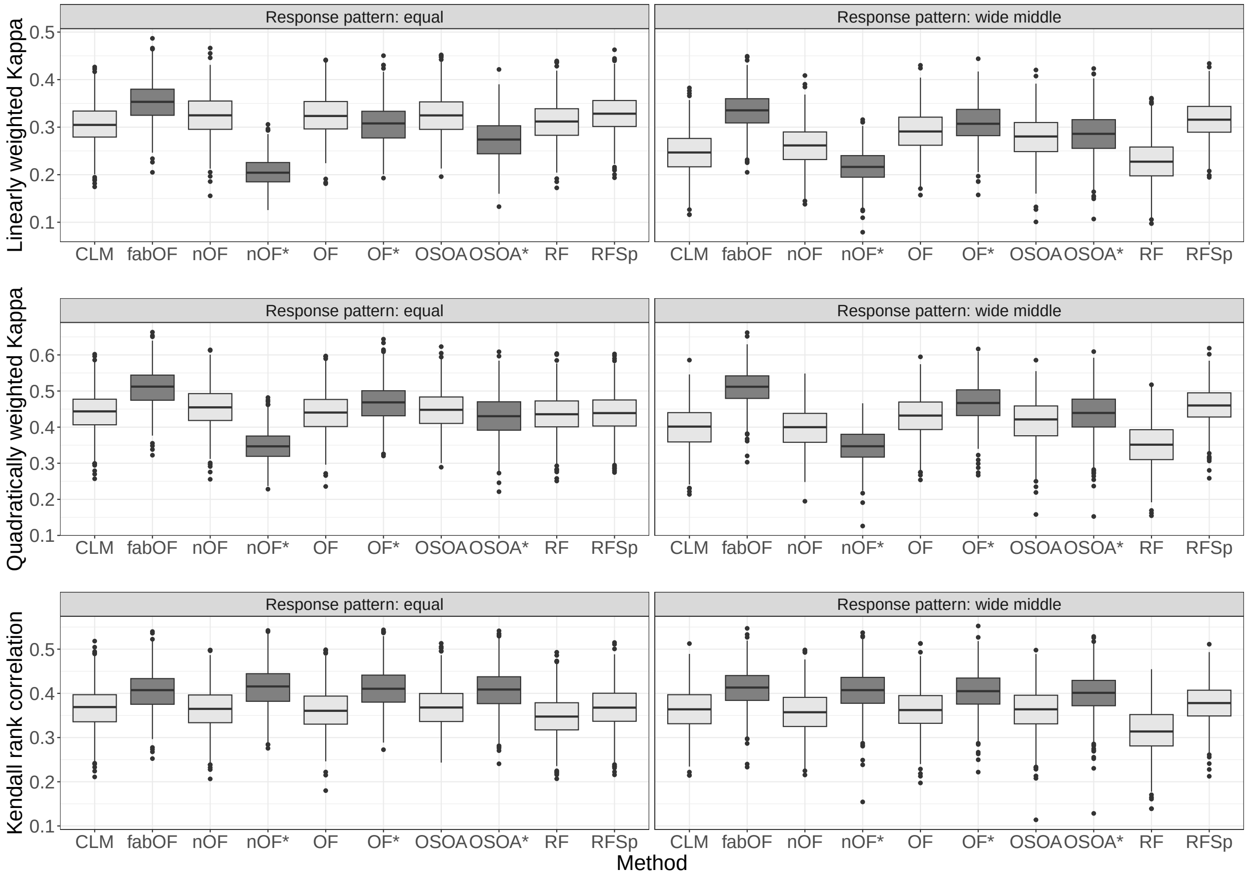

Figure 17

Predictive performance of all methods and modifications for data simulated from DGP 3 with $n = 750$, $k = 7$ and random sampling for both response category distribution patterns. Approaches using AFTA prediction indicated through dark grey colored boxplots with additional asterisk indicating modification of existing method.

Note: CLM: Cumulative Link Model (proportional odds), fabOF: Frequency-Adjusted Borders Ordinal Forest, nOF: naive Ordinal Forest, OF: Ordinal Forest, OSOA: Ordinal Score Optimization Algorithm, RF: Random Forest, RFSp: Split-based Ordinal Forest.

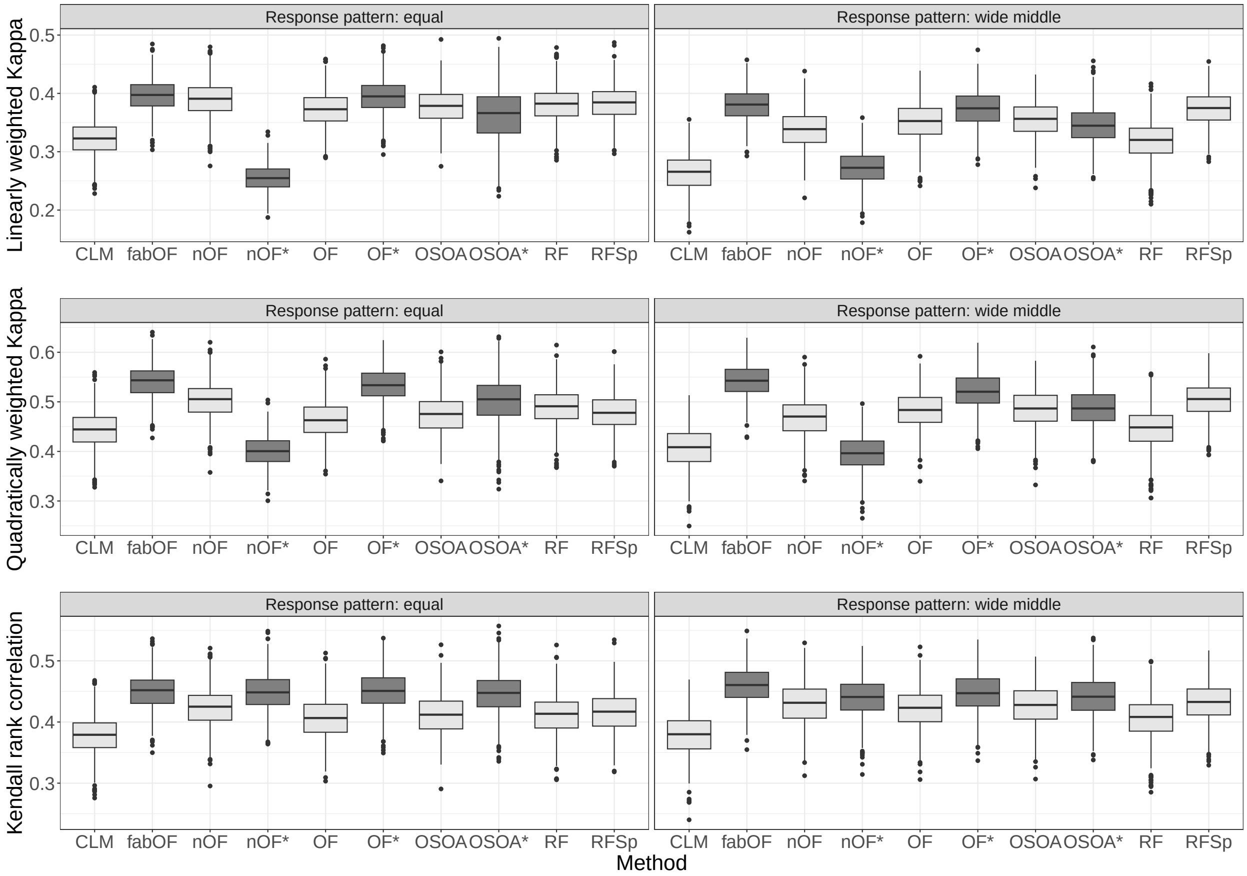

Figure 18

Predictive performance of all methods and modifications for data simulated from DGP 3 with $n = 1500$*,* $k = 5$ *and stratified sampling for both response category distribution patterns. Approaches using AFTA prediction indicated through dark grey colored boxplots with additional asterisk indicating modification of existing method.*

Note: CLM: Cumulative Link Model (proportional odds), fabOF: Frequency-Adjusted Borders Ordinal Forest, nOF: naive Ordinal Forest, OF: Ordinal Forest, OSOA: Ordinal Score Optimization Algorithm, RF: Random Forest, RFSp: Split-based Ordinal Forest.

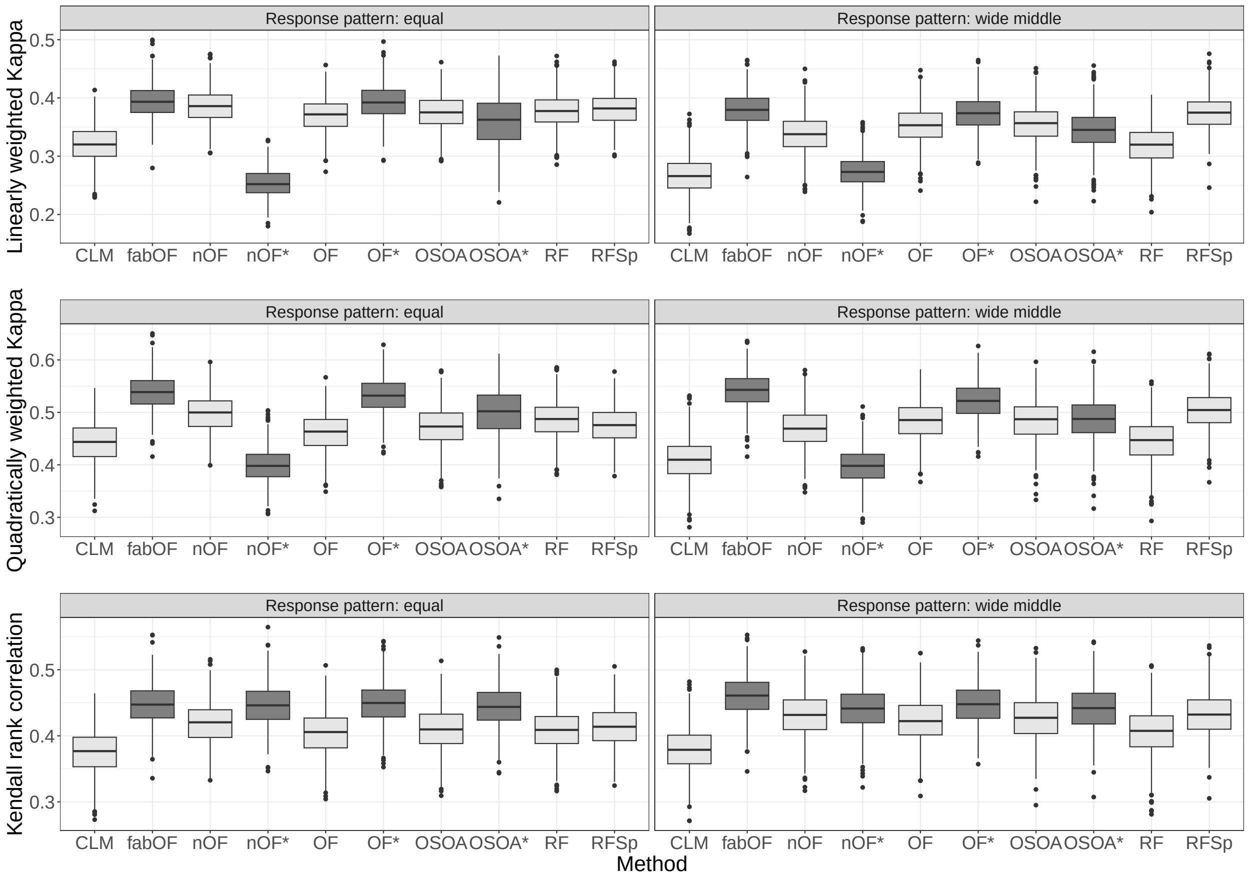

Figure 19

Predictive performance of all methods and modifications for data simulated from DGP 3 with $n = 1500$, $k = 5$ and random sampling for both response category distribution patterns. Approaches using AFTA prediction indicated through dark grey colored boxplots with additional asterisk indicating modification of existing method.

Note: CLM: Cumulative Link Model (proportional odds), fabOF: Frequency-Adjusted Borders Ordinal Forest, nOF: naive Ordinal Forest, OF: Ordinal Forest, OSOA: Ordinal Score Optimization Algorithm, RF: Random Forest, RFSp: Split-based Ordinal Forest.

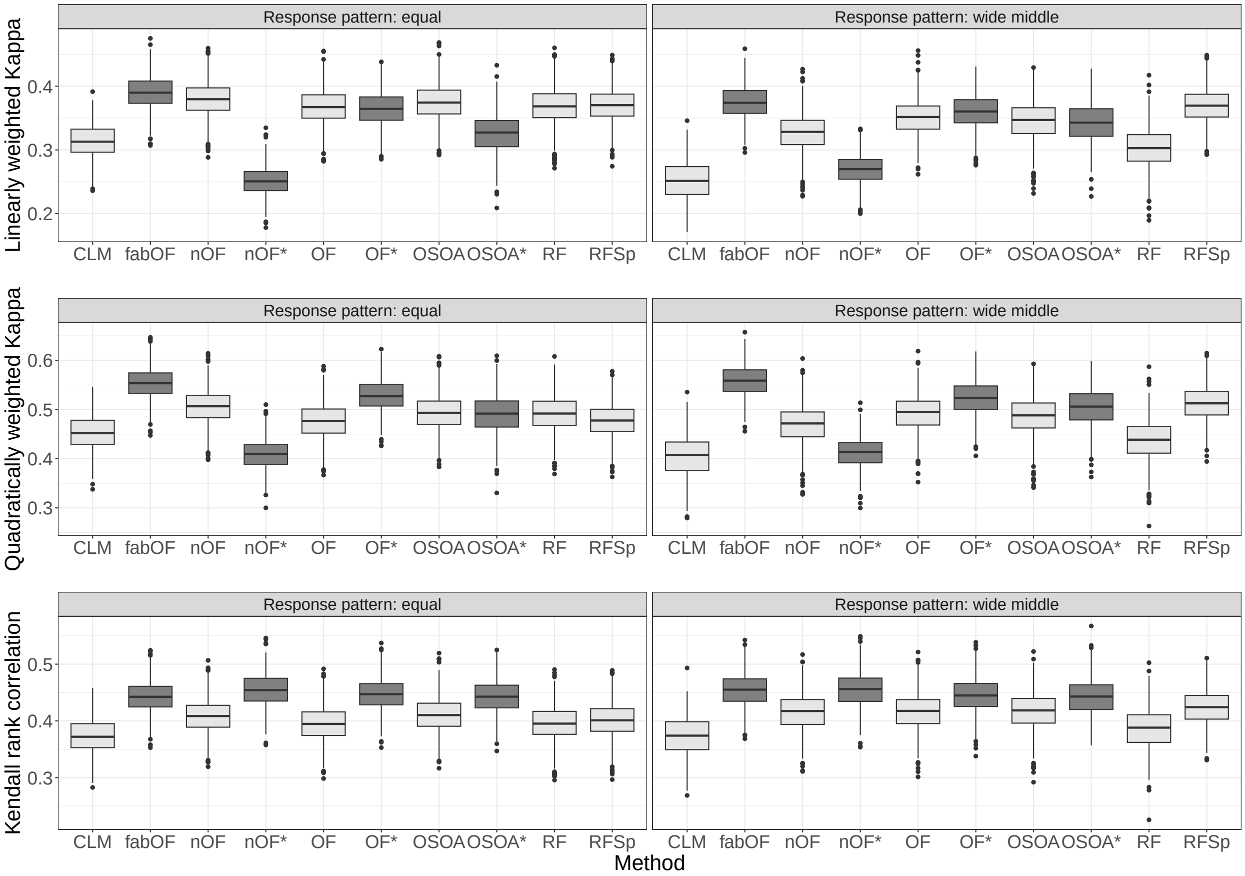

Figure 20

Predictive performance of all methods and modifications for data simulated from DGP 3 with $n = 1500$, $k = 7$ and stratified sampling for both response category distribution patterns. Approaches using AFTA prediction indicated through dark grey colored boxplots with additional asterisk indicating modification of existing method.

Note: CLM: Cumulative Link Model (proportional odds), fabOF: Frequency-Adjusted Borders Ordinal Forest, nOF: naive Ordinal Forest, OF: Ordinal Forest, OSOA: Ordinal Score Optimization Algorithm, RF: Random Forest, RFSp: Split-based Ordinal Forest.

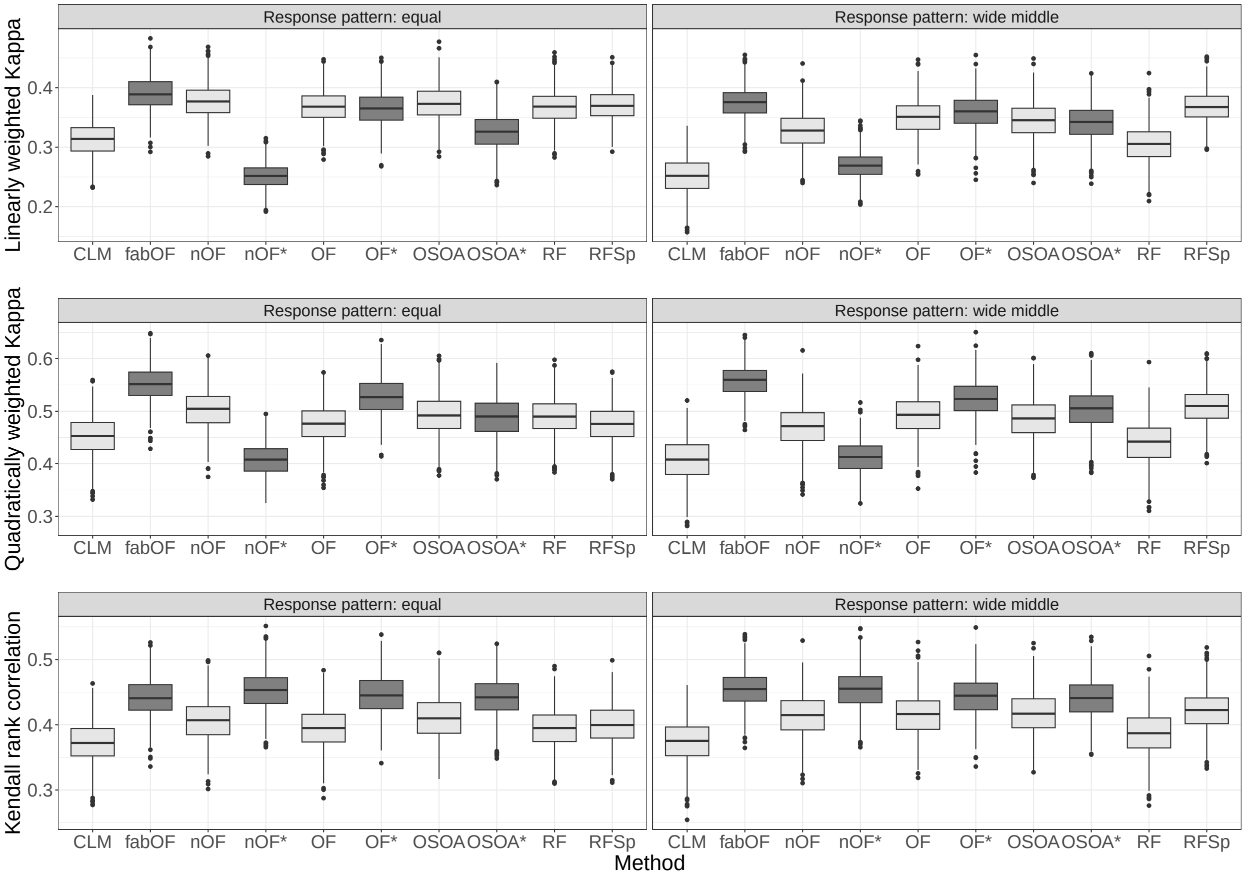

Figure 21

Predictive performance of all methods and modifications for data simulated from DGP 3 with $n = 1500$, $k = 7$ and random sampling for both response category distribution patterns. Approaches using AFTA prediction indicated through dark grey colored boxplots with additional asterisk indicating modification of existing method.

Note: CLM: Cumulative Link Model (proportional odds), fabOF: Frequency-Adjusted Borders Ordinal Forest, nOF: naive Ordinal Forest, OF: Ordinal Forest, OSOA: Ordinal Score Optimization Algorithm, RF: Random Forest, RFSp: Split-based Ordinal Forest.

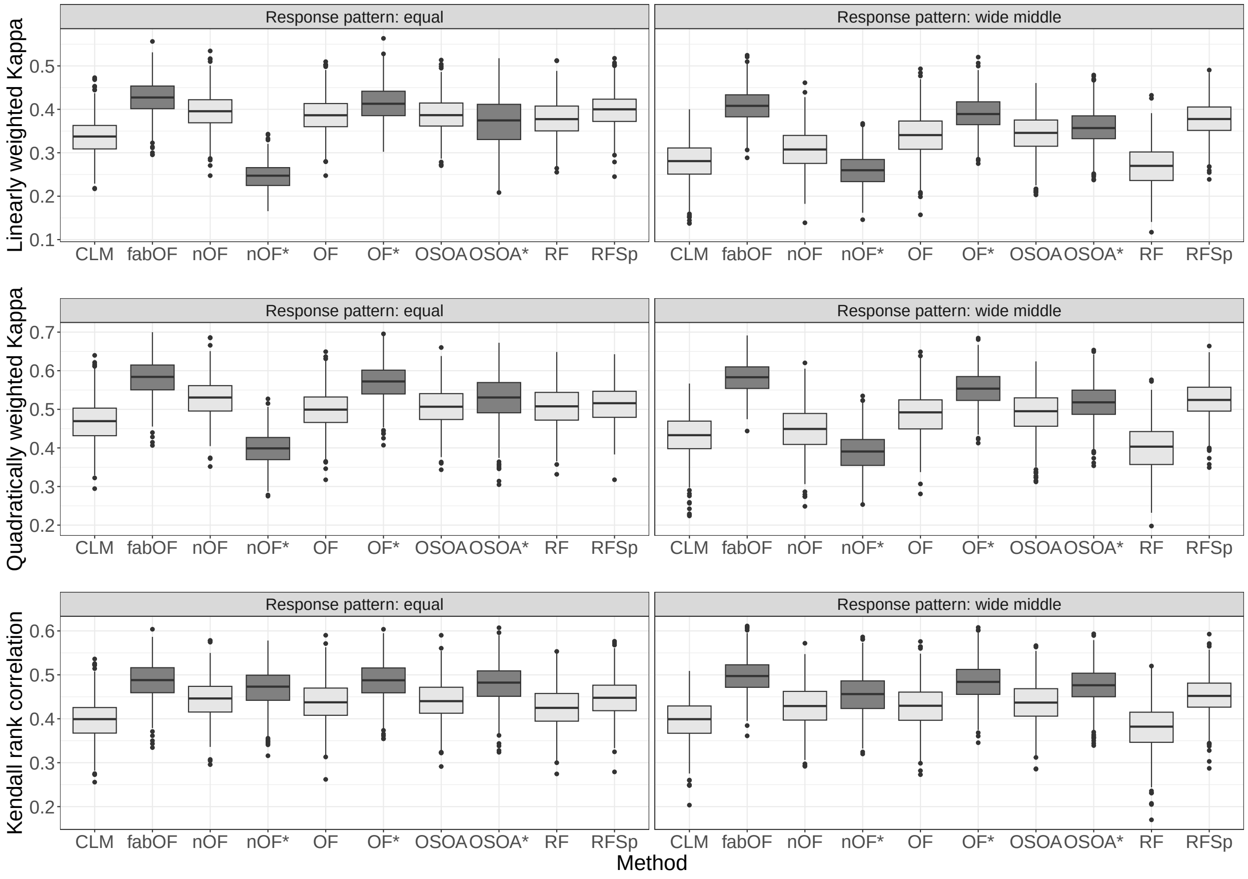

Figure 22

Predictive performance of all methods and modifications for data simulated from DGP 4 with $n = 750$, $k = 5$ and random sampling for both response category distribution patterns. Approaches using AFTA prediction indicated through dark grey colored boxplots with additional asterisk indicating modification of existing method.

Note: CLM: Cumulative Link Model (proportional odds), fabOF: Frequency-Adjusted Borders Ordinal Forest, nOF: naive Ordinal Forest, OF: Ordinal Forest, OSOA: Ordinal Score Optimization Algorithm, RF: Random Forest, RFSp: Split-based Ordinal Forest.

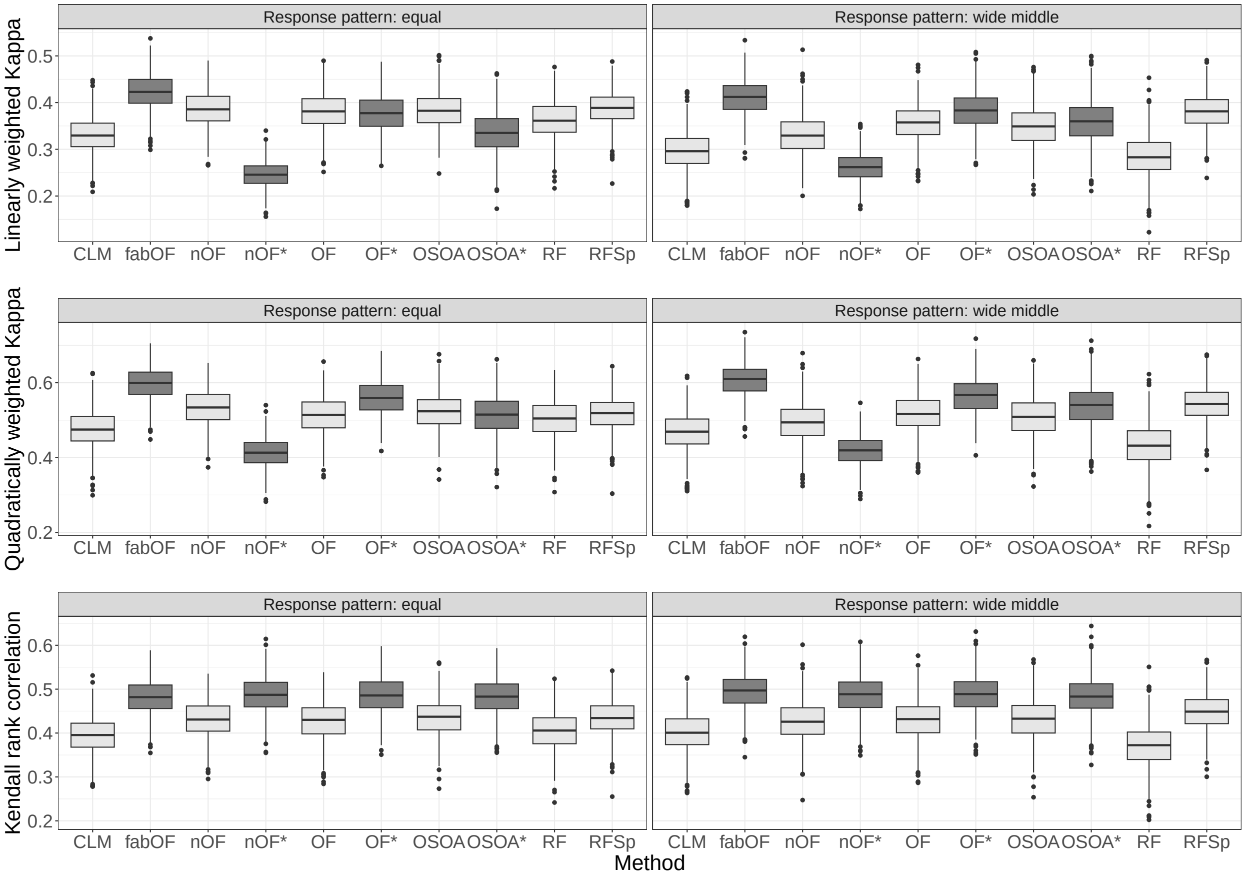

Figure 23

Predictive performance of all methods and modifications for data simulated from DGP 4 with $n = 750$, $k = 7$ and stratified sampling for both response category distribution patterns. Approaches using AFTA prediction indicated through dark grey colored boxplots with additional asterisk indicating modification of existing method.

Note: CLM: Cumulative Link Model (proportional odds), fabOF: Frequency-Adjusted Borders Ordinal Forest, nOF: naive Ordinal Forest, OF: Ordinal Forest, OSOA: Ordinal Score Optimization Algorithm, RF: Random Forest, RFSp: Split-based Ordinal Forest.

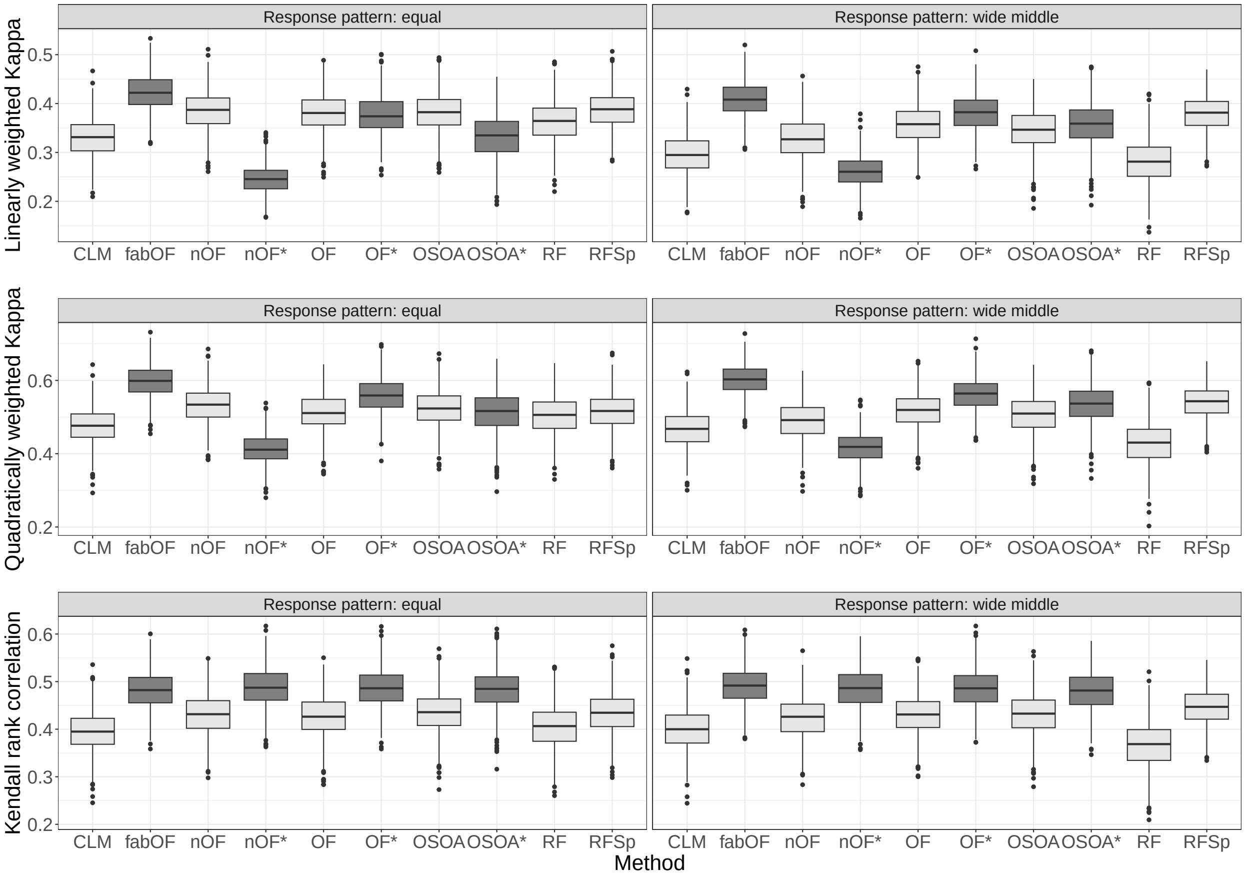

Figure 24

Predictive performance of all methods and modifications for data simulated from DGP 4 with $n = 750$, $k = 7$ *and random sampling for both response category distribution patterns. Approaches using AFTA prediction indicated through dark grey colored boxplots with additional asterisk indicating modification of existing method.*

Note: CLM: Cumulative Link Model (proportional odds), fabOF: Frequency-Adjusted Borders Ordinal Forest, nOF: naive Ordinal Forest, OF: Ordinal Forest, OSOA: Ordinal Score Optimization Algorithm, RF: Random Forest, RFSp: Split-based Ordinal Forest.

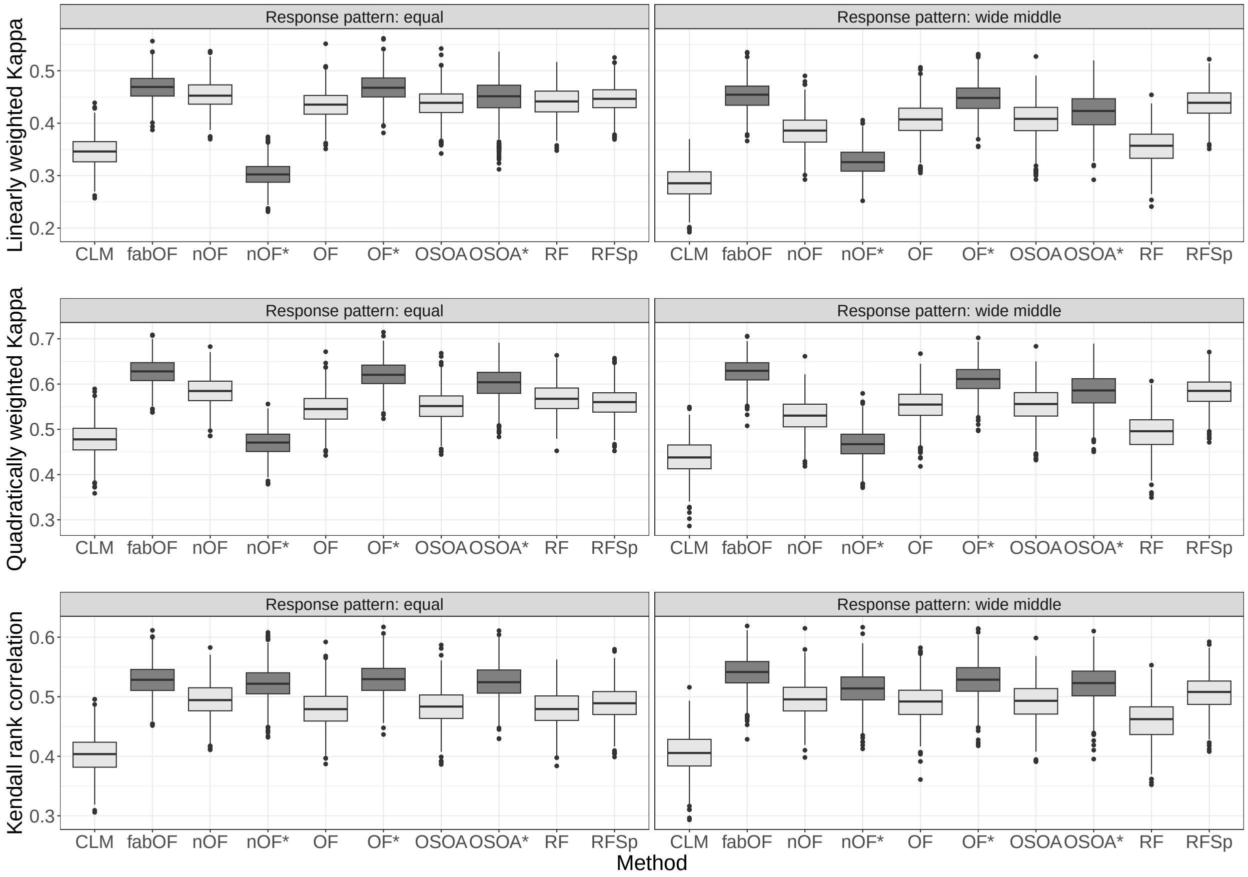

Figure 25

Predictive performance of all methods and modifications for data simulated from DGP 4 with $n = 1500$, $k = 5$ and stratified sampling for both response category distribution patterns. Approaches using AFTA prediction indicated through dark grey colored boxplots with additional asterisk indicating modification of existing method.

Note: CLM: Cumulative Link Model (proportional odds), fabOF: Frequency-Adjusted Borders Ordinal Forest, nOF: naive Ordinal Forest, OF: Ordinal Forest, OSOA: Ordinal Score Optimization Algorithm, RF: Random Forest, RFSp: Split-based Ordinal Forest.

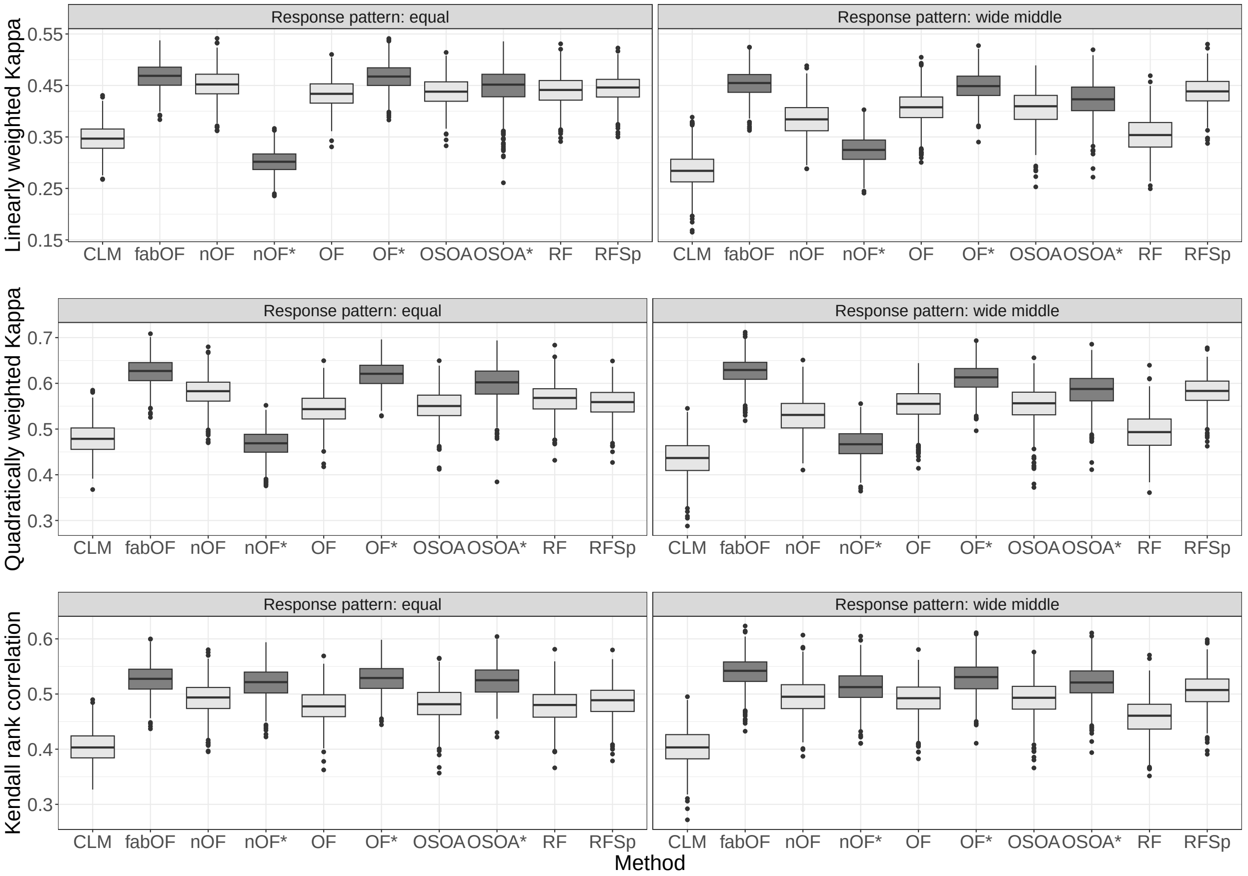

Figure 26

Predictive performance of all methods and modifications for data simulated from DGP 4 with $n = 1500$, $k = 5$ and random sampling for both response category distribution patterns. Approaches using AFTA prediction indicated through dark grey colored boxplots with additional asterisk indicating modification of existing method.

Note: CLM: Cumulative Link Model (proportional odds), fabOF: Frequency-Adjusted Borders Ordinal Forest, nOF: naive Ordinal Forest, OF: Ordinal Forest, OSOA: Ordinal Score Optimization Algorithm, RF: Random Forest, RFSp: Split-based Ordinal Forest.

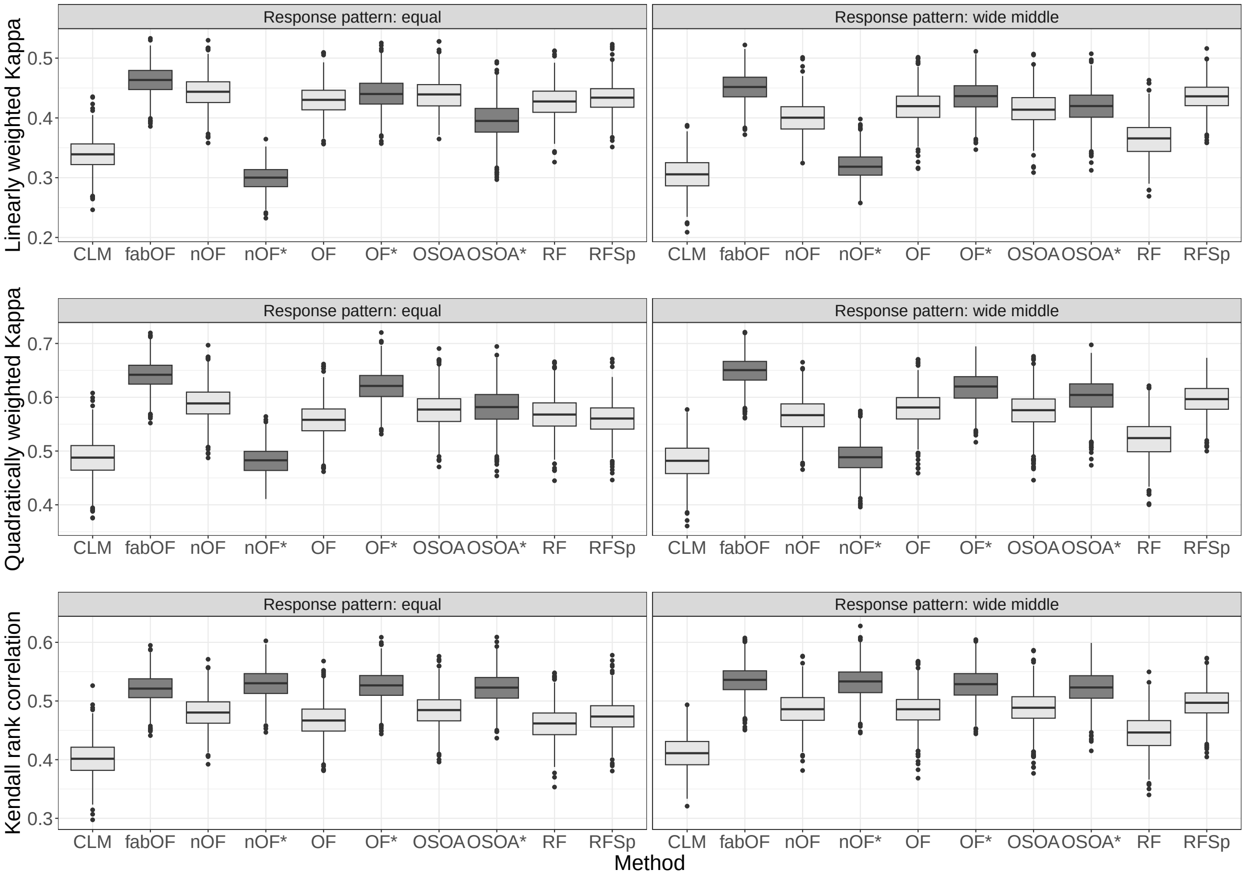

Figure 27

Predictive performance of all methods and modifications for data simulated from DGP 4 with $n = 1500$, $k = 7$ and stratified sampling for both response category distribution patterns. Approaches using AFTA prediction indicated through dark grey colored boxplots with additional asterisk indicating modification of existing method.

Note: CLM: Cumulative Link Model (proportional odds), fabOF: Frequency-Adjusted Borders Ordinal Forest, nOF: naive Ordinal Forest, OF: Ordinal Forest, OSOA: Ordinal Score Optimization Algorithm, RF: Random Forest, RFSp: Split-based Ordinal Forest.

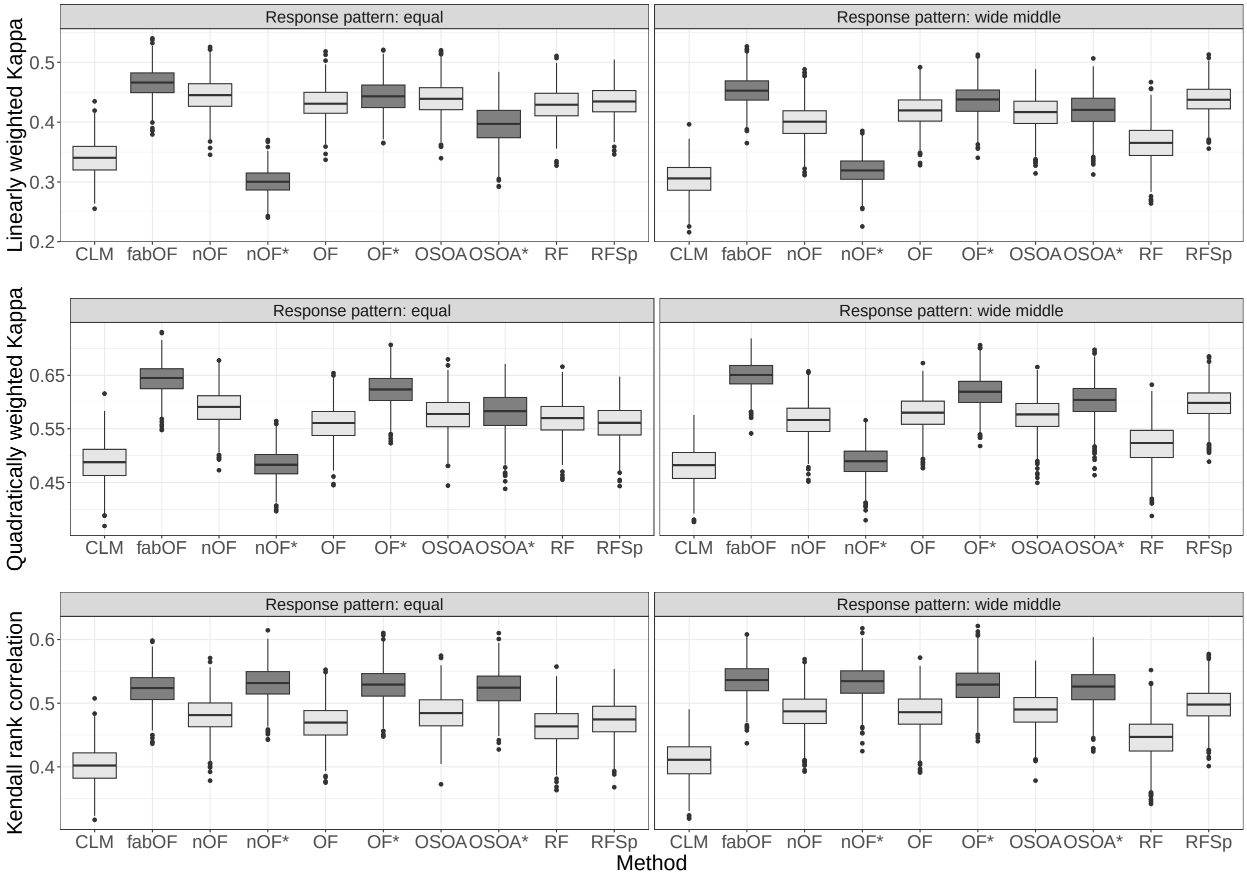

Figure 28

Predictive performance of all methods and modifications for data simulated from DGP 4 with $n = 1500$, $k = 7$ and random sampling for both response category distribution patterns. Approaches using AFTA prediction indicated through dark grey colored boxplots with additional asterisk indicating modification of existing method.

Note: CLM: Cumulative Link Model (proportional odds), fabOF: Frequency-Adjusted Borders Ordinal Forest, nOF: naive Ordinal Forest, OF: Ordinal Forest, OSOA: Ordinal Score Optimization Algorithm, RF: Random Forest, RFSp: Split-based Ordinal Forest.

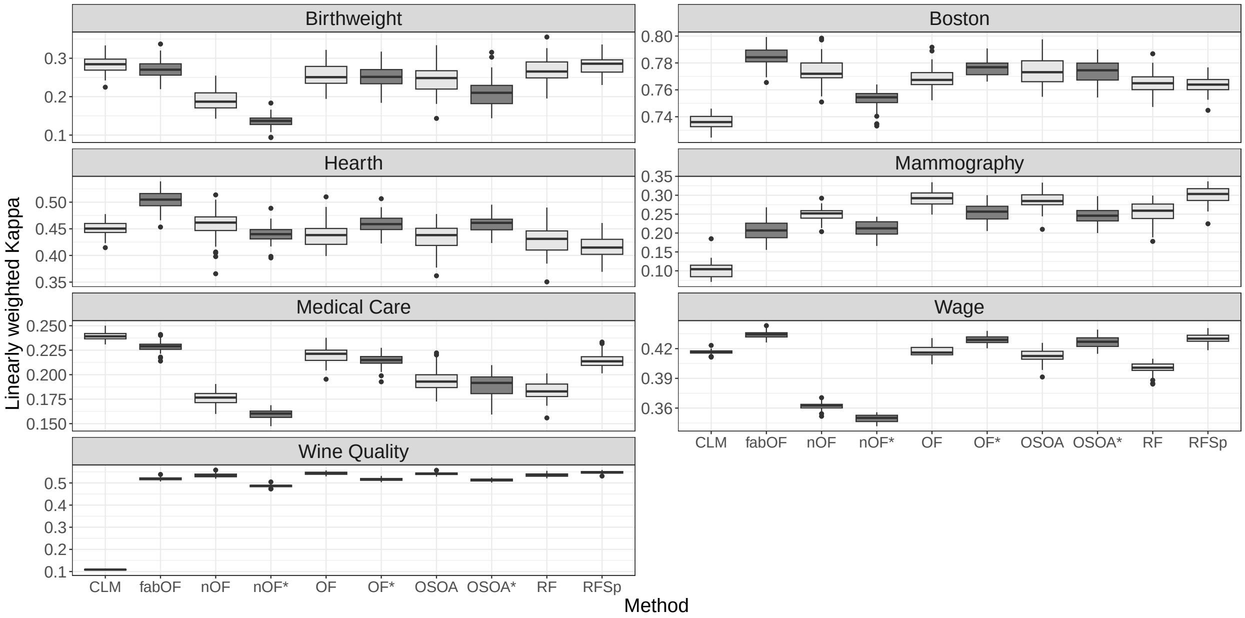

Figure 29

Linearly weighted Kappa values achieved by methods and modifications for additional real datasets as used in Buczak et al. (2024). Approaches using AFTA prediction indicated through dark grey colored boxplots with additional asterisk indicating modification of existing method.

Note: CLM: Cumulative Link Model (proportional odds), fabOF: Frequency-Adjusted Borders Ordinal Forest, nOF: naive Ordinal Forest, OF: Ordinal Forest, OSOA: Ordinal Score Optimization Algorithm, RF: Random Forest, RFSp: Split-based Ordinal Forest.

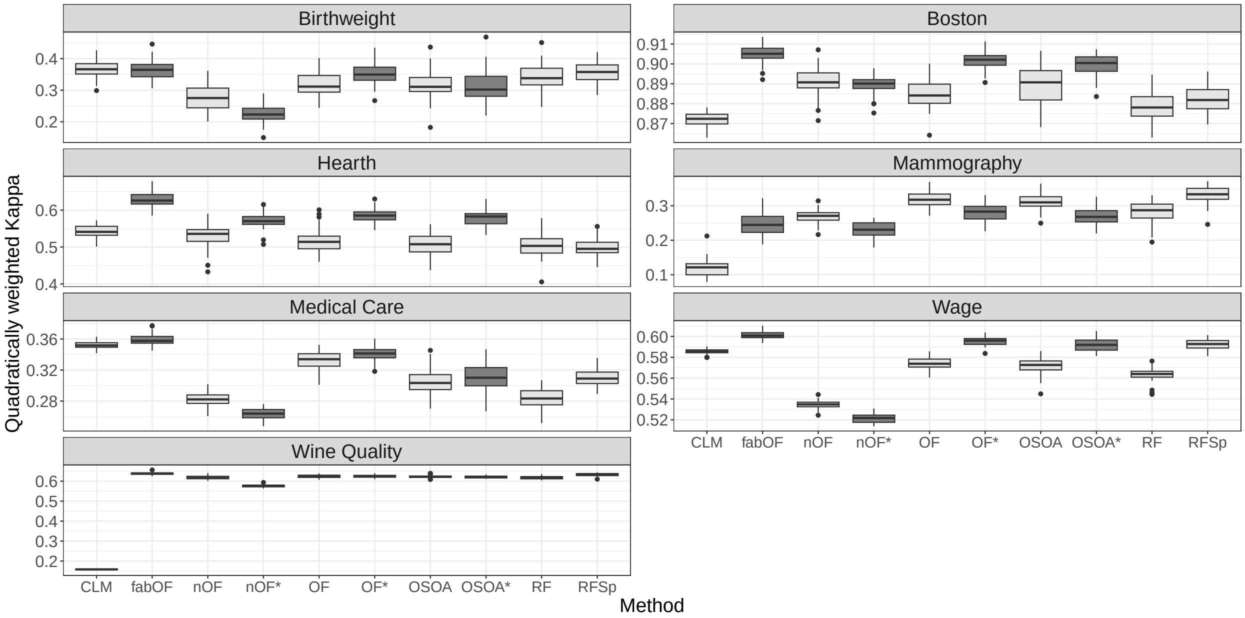

Figure 30

Quadratically weighted Kappa values achieved by methods and modifications for additional real datasets as used in Buczak et al. (2024). Approaches using AFTA prediction indicated through dark grey colored boxplots with additional asterisk indicating modification of existing method.

Note: CLM: Cumulative Link Model (proportional odds), fabOF: Frequency-Adjusted Borders Ordinal Forest, nOF: naive Ordinal Forest, OF: Ordinal Forest, OSOA: Ordinal Score Optimization Algorithm, RF: Random Forest, RFSp: Split-based Ordinal Forest.

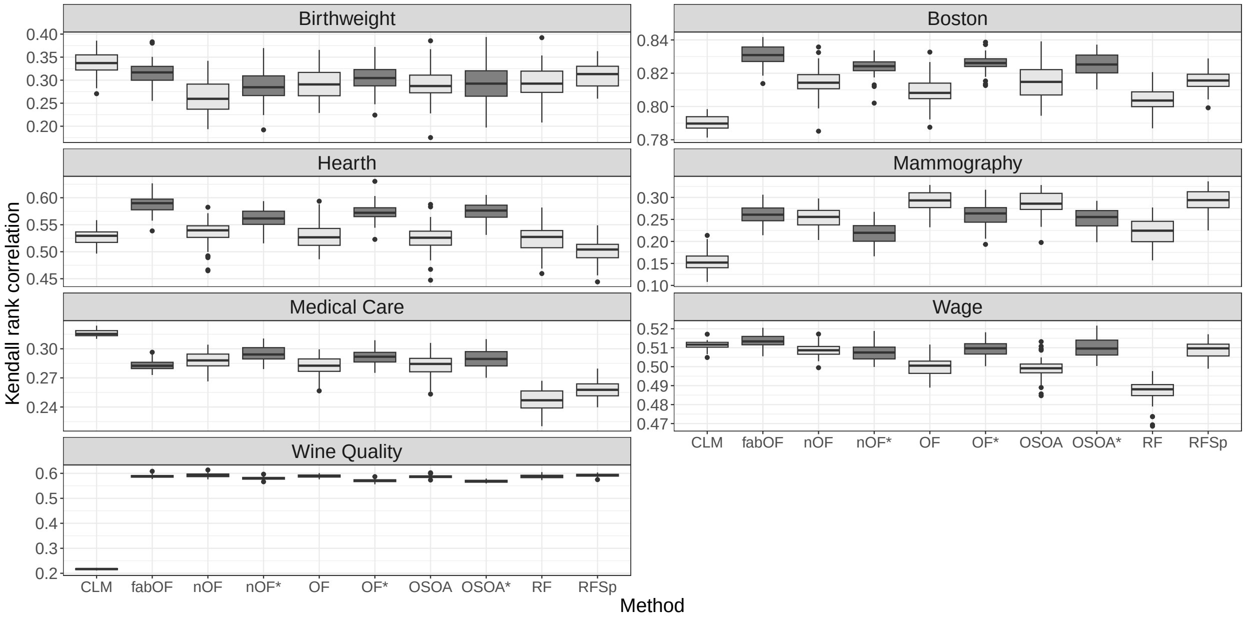

Figure 31

Kendall rank correlation values achieved by methods and modifications for additional real datasets as used in Buczak et al. (2024). Approaches using AFTA prediction indicated through dark grey colored boxplots with additional asterisk indicating modification of existing method.

Note: CLM: Cumulative Link Model (proportional odds), fabOF: Frequency-Adjusted Borders Ordinal Forest, nOF: naive Ordinal Forest, OF: Ordinal Forest, OSOA: Ordinal Score Optimization Algorithm, RF: Random Forest, RFSp: Split-based Ordinal Forest.

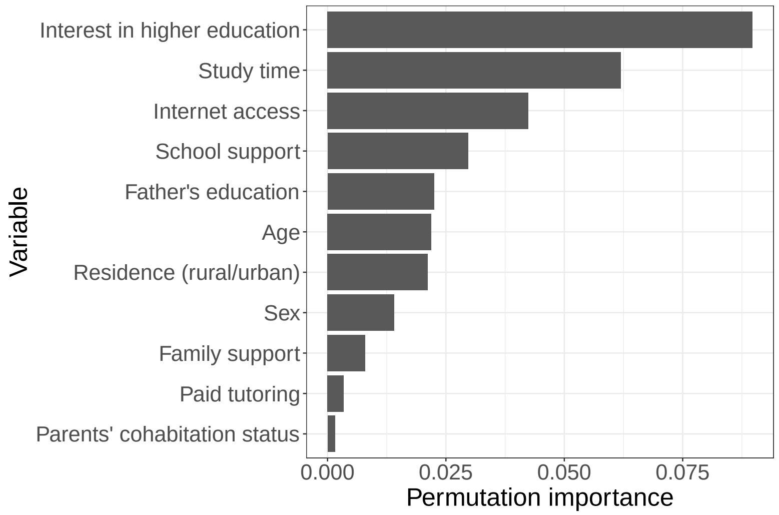

Figure 32

Permutation variable importance for student performance data when excluding mother's education from the model.
